# Dynamics of Influenza A (H5N1) virus protein sequence diversity

**DOI:** 10.7717/peerj.7954

**Published:** 2020-05-27

**Authors:** Hadia Syahirah Abd Raman, Swan Tan, Joseph Thomas August, Asif M. Khan

**Affiliations:** 1 School of Data Sciences, Perdana University, Serdang, Selangor, Malaysia; 2 Institute for Immunology and Informatics, University of Rhode Island, Providence, RI, United States of America; 3 School of Medicine, Johns Hopkins University, Baltimore, MD, United States of America; 4 Beykoz Institute of Life Sciences and Biotechnology, Bezmialem Vakif University, Beykoz, Istanbul, Turkey

**Keywords:** H5N1, Influenza virus, Sequence diversity, Variants, Fitness-selection, Dynamics

## Abstract

**Background:**

Influenza A (H5N1) virus is a global concern with potential as a pandemic threat. High sequence variability of influenza A viruses is a major challenge for effective vaccine design. A continuing goal towards this is a greater understanding of influenza A (H5N1) proteome sequence diversity in the context of the immune system (antigenic diversity), the dynamics of mutation, and effective strategies to overcome the diversity for vaccine design.

**Methods:**

Herein, we report a comprehensive study of the dynamics of H5N1 mutations by analysis of the aligned overlapping nonamer positions (1–9, 2–10, etc.) of more than 13,000 protein sequences of avian and human influenza A (H5N1) viruses, reported over at least 50 years. Entropy calculations were performed on 9,408 overlapping nonamer position of the proteome to study the diversity in the context of immune system. The nonamers represent the predominant length of the binding cores for peptides recognized by the cellular immune system. To further dissect the sequence diversity, each overlapping nonamer position was quantitatively analyzed for four patterns of sequence diversity motifs: index, major, minor and unique.

**Results:**

Almost all of the aligned overlapping nonamer positions of each viral proteome exhibited variants (major, minor, and unique) to the predominant index sequence. Each variant motif displayed a characteristic pattern of incidence change in relation to increased total variants. The major variant exhibited a restrictive pyramidal incidence pattern, with peak incidence at 50% total variants. Post this peak incidence, the minor variants became the predominant motif for majority of the positions. Unique variants, each sequence observed only once, were present at nearly all of the nonamer positions. The diversity motifs (index and variants) demonstrated complex inter-relationships, with motif switching being a common phenomenon. Additionally, 25 highly conserved sequences were identified to be shared across viruses of both hosts, with half conserved to several other influenza A subtypes.

**Discussion:**

The presence of distinct sequences (nonatypes) at nearly all nonamer positions represents a large repertoire of reported viral variants in the proteome, which influence the variability dynamics of the viral population. This work elucidated and provided important insights on the components that make up the viral diversity, delineating inherent patterns in the organization of sequence changes that function in the viral fitness-selection. Additionally, it provides a catalogue of all the mutational changes involved in the dynamics of H5N1 viral diversity for both avian and human host populations. This work provides data relevant for the design of prophylactics and therapeutics that overcome the diversity of the virus, and can aid in the surveillance of existing and future strains of influenza viruses.

## Introduction

Influenza A viruses are a major public health problem worldwide (Szewczyk, Bieńkowska-szewczyk & Król, 2014). Infections of humans commonly occur every year (Webster, 1998; Fiers et al., 2004), sometimes as a fatal illness. A major concern is the emergence of highly infectious subtype strains that lead to global pandemics. Major influenza pandemics have been recorded in the past, starting with the deadly “Spanish Flu” of 1918 that was caused by the H1N1 subtype, with an estimated 50 million fatalities (Taubenberger & Morens, 2006). The H2N2 subtype “Asian influenza” in 1957, H3N2 subtype “Hong Kong Flu” in 1968 and H1N1 “swine flu” in 2009, were less severe but estimated to have resulted in 100,000 fatalities (Cox, Tamblyn & Tam, 2003; Uyeki, 2008; Shrestha et al., 2011). Without effective means to prevent or control influenza infection by new strains, there is the looming threat of the next influenza pandemic (Chaturvedi, 2009).

H5N1 subtype of influenza A virus (H5N1 IAV) is a global concern with pandemic potential (classified as phase 3 alert by the World Health Organization; World Health Organization, 2013). A series of outbreaks of the virus have been reported, the first of which emerged in 1997 in Hong Kong and infected 18 patients, six of whom died (Neumann et al., 2005; World Health Organization, 2014). There were 860 confirmed human cases of avian H5N1 IAV and 454 deaths reported by WHO as of December 13, 2018 (World Health Organization, 2018). The annual influenza vaccine formulation is not designed for prevention of H5N1 infection (World Health Organization, 2012) and currently, there is no effective vaccine against the virus that is ready for widespread use.

The genome of IAV comprises of eight negative-sense, single-stranded RNA segments. Segments 1, and 4 to 6 encode for a single protein each (PB2, HA, NP, and NA, respectively); Segments 2, 3 and 7 encode for two proteins each (PB1 and PB1-F2, PA and PA-X, M1 and M2, respectively), while segment 8 encodes for two non-structural proteins (NS1 and NS2). Each RNA segment of the genome is wrapped around nucleoprotein (NP) monomers and is associated with polymerase acidic protein (PA) and polymerase basic proteins (PB1 and PB2), forming a ribonucleoprotein (RNP) complex, which is important for viral transcription and replication (Taubenberger & Kash, 2010; Noda & Kawaoka, 2010). The RNP complexes are contained in a spherical lipid envelope lined by M1 proteins and embedded with surface glycoproteins, hemagglutinin (HA) and neuraminidase (NA), and the ion-channel protein, M2. HA protein is key for the entry of the virus through binding to the sialic acid receptor of the host, while NA protein functions in releasing the virus progeny out of the host cell (Khanna et al., 2014). The high sequence diversity exhibited by the virus makes vaccine development a challenging task (Dunston, Herbert & Griffiths, 2015). The diversity is achieved through the accumulation of point mutations (antigenic drift) across the segmented genome of the RNA virus, which encodes for 12 proteins, and the exchange of gene segments between two different strains (antigenic shift). The gradual antigenic drift is caused by the lack of proofreading fidelity by the viral polymerase, thus resulting in a high rate of mutation, approximately one per genome per replication (Drake et al., 1998). Antigenic drift can lead to the establishment of viral variants that escape immune recognition and is associated with frequent epidemics (Potter, 2001). Antigenic shift, in contrast, occurs abruptly, producing new “immune-escape” viral variant strains that can potentially give rise to pandemics (Szewczyk, Bieńkowska-szewczyk & Król, 2014). The ongoing evolution and diversification of the H5N1 IAV challenges the conventional method of vaccine formulation, which is time-consuming, of limited production and strain-specific. Thus, it is crucial to understand the diversity and the dynamics of viral sequence change to aid in the design of vaccine or development of therapeutics against the virus.

Heiny et al. (2007) had performed a large-scale analyses on the evolutionary diversity of several influenza A virus subtypes in the context of the immune system and reported that H5N1 has a high mutational variability for proteins of both avian and human strains. A continuing goal is a greater understanding of H5N1 IAV proteome sequence diversity, the dynamics of mutation, and effective strategies to overcome the diversity for vaccine design. Thus, a more detailed, quantitative analyses of H5N1 IAV sequence changes is necessary.

Herein, we address the issue through a comprehensive quantitative bioinformatics analyses of both avian and human H5N1 IAV protein sequences reported in public databases. Influenza A virus sequence data is publicly available in specialized repositories, such as the Influenza Research Database (http://www.fludb.org) (Squires et al., 2012). Bioinformatics methodologies are necessary to manage and mine large and complex biological datasets. In order to analyze the diversity in the context of the immune system (antigenic diversity), the bioinformatics analyses were performed on aligned nonamer sequences, overlapping by eight residues (1–9, 2–10, etc.), where each of the sequences at the nonamer positions represents a potential ligand of human leukocyte antigens (HLAs) and T-cell receptors (TCRs) (Hu et al., 2013). This proteome-wide, quantitative characterization of H5N1 IAV sequence change provides data relevant to the design of therapeutics that overcome the diversity of the virus, besides additional possible utility of the data for diagnostics and surveillance purposes.

## Materials & Methods

### Proteome sequence data collection and processing

Avian and human H5N1 IAV protein sequences, both partial and full-length, and the corresponding metadata were retrieved from the publicly available Influenza Research Database (http://www.fludb.org; as of January 2013) (Squires et al., 2012). Partial sequences were included for a comprehensive collection of the virus sequence data. The collected data were classified according to the 12 influenza A proteins (PB2, PB1, PB1-F2, PA, PA-X, HA, NP, NA, M1, M2, NS2, NS1) for each host, avian and human. These hosts were chosen because they cover the majority of the reported influenza A virus sequences. The classified data were manually checked and cleaned of any irrelevant sequences.

The viral protein sequences of each host were deduplicated by use of JalView (Waterhouse et al., 2009) to minimize bias and then aligned by use of the multiple sequence alignment program Clustal Omega (Sievers et al., 2011). All alignments were manually checked and corrected for any inconsistencies, empty columns, gaps and misalignments. Alignment positions that contained 95% or more gaps (insertions or deletions) were removed from the dataset as they had reduced statistical support and can result in artificial diversity measures (Khan et al., 2008). Alignment positions described in this manuscript are based on the alignment generated from the data used herein.

### Mapping the proteome sequence diversity

Shannon’s entropy (Shannon, 1948) was used to provide an overview of the H5N1 IAV proteome sequence diversity. A sliding k-mer window approach was utilized to assess the diversity in the context of cellular immune response. A k-mer size of nine was chosen because it represents the predominant length of peptides recognized by human leukocyte antigens (HLAs) and T-cell receptor (TCR) binding domains (Rammensee, 1995; Heiny et al., 2007). The diversity at each of the overlapping 9-mer (nonamer) positions across the length of the protein alignments was measured by use of Shannon’s entropy formula (Shannon, 1948), modified for nonamers (Khan et al., 2006; Khan et al., 2008; Heiny et al., 2007; Koo et al., 2009; Miotto et al., 2010; Hu et al., 2013). Entropy, *H(x)* at a given nonamer position *x* in the alignment was calculated as follows:

\begin{eqnarray*}H \left( x \right) =-\sum _{i=1}^{n(x)}p \left( i,x \right) {\log \nolimits }_{2}p(i,x) \end{eqnarray*}

where *p(i,x)* is the probability of a given nonamer sequence *i* at nonamer position *x*. The entropy value, *H* at a given position increases with an increase in total number of distinct nonamer sequences, *n(x)*. It is also affected by the relative probability of the sequences, such that it decreases when one sequence is significantly dominant at the position (i.e., the position is conserved). The entropy value represents the diversity of the sequences at a given nonamer position, ranging from 0, a completely conserved position, to a theoretical maximum of 39, where all possible nonamer combinations are observed.

Entropy measure is also dependent on the number of sequences in the alignment. Thus, the entropy value was corrected for size bias through a statistical adjustment as described in Khan et al. (2008). Any nonamer sequence with a gap (-) or an ambiguous character (B, asparagine or aspartic acid; J, leucine or isoleucine; X, unspecified or unknown amino acid; and Z, glutamine or glutamic acid) was removed from the analyses.

### Quantifying the dynamics of sequence change

All sequences at each of the nonamer positions in the protein alignments were quantified and ranked based on the incidences of distinct sequences (as part of diversity motifs) present at the position, as described in Hu et al. (2013) (Fig. S1) by use of an in-house Perl program. The patterns of the dynamics of sequence change and the frequency distribution of each of the diversity motifs for the avian and the human viruses were plotted using the Ggplot2 package (Wickham, 2016) or R (R Core Team, 2017) and studied in relation to increased total variants. The occurrence of motif switching as previously indicated by Hu et al. (2013), where fitness change of amino acids resulted in a motif change, was evaluated for both the human and avian viral proteomes.

### Immune relevance of motif switching

Motif switching positions were studied for their immune-relevance by use of T-cell epitope and B-cell antigenicity prediction tools (Dhanda et al., 2017; Hasan et al., 2019). Only index switching positions were analyzed for this, given the relevance of the index sequence for vaccine target selection. There were only a total of nine such positions across both the avian and human viral proteomes; one was not analyzed due to low sequence number support. The index sequence at a given index switching position was the major variant in the preceding position, while the major variant at the given position was the preceding index sequence. Thus, predicting the immune relevance of the index and the major variant at the index switching position alone would be indicative of a loss or gain of an epitope, relative to each other. NetCTL (Larsen et al., 2007) was used for prediction of HLA class I supertype-restricted T-cell epitopes (12 supertypes). NetMHCII (Jensen et al., 2018) was used for class II DR supertypes by predicting for 15 representative HLA alleles (DRB1_0101, DRB1_0701, DRB1_0901, DRB1_1101, DRB1_1201, DRB1_1501, DRB5_0101, DRB1_0401, DRB1_0405, DRB1_0802, DRB1_0301, DRB1_1301, DRB3_0101, DRB3_0202, DRB4_0103, DRB4_0101); a peptide was considered as putative epitope if prediction was positive for at least half of the alleles of the respective DR supertype. Bepipred 2.0 (Jespersen et al., 2017), accessed through Immune Epitope Database (IEDB), and LBtope (Singh, Ansari & Raghava, 2013) were used for prediction of linear B-cell epitopes because they were among the latest benchmarked tools available. The respective default thresholds of these two tools were used to evaluate the antigenicity of the nonamer peptides (index and major) at the index switching positions.

### Distribution of conserved and variable nonamer positions in avian and human influenza A (H5N1) and immune relevance

The index sequence incidences across all the nonamer positions were categorized into three levels of conservation, to study the proteome distribution of conserved and variable sequences: 1) Highly conserved (index incidence ≥ 90%), 2) Highly diverse (index incidence ≤ 20%), and 3) Mixed-variable (index incidence between 90% and 20%). The dynamics of T-cell epitopes for both avian and human H5N1 IAV were studied by randomly selecting three nonamer positions for each conservation level, which corresponded to both hosts –highly conserved (PA: 297–305, PB1: 673–681, and PA: 244–252), highly diverse (only one position qualified for this level; HA: 170–178) and mixed-variable (PB2: 361–369, PA: 59–67, HA: 497–505). The immune relevance of all the unique nonamer sequences (nonatypes) at the selected positions were studied by use of NetCTL (Larsen et al., 2007) for prediction of HLA class I supertype-restricted T-cell epitopes (12 supertypes). The pattern of the predictions was compared between the corresponding positions of the two hosts.

### Identification of highly conserved sequences

Highly conserved sequences, with extremely low entropy, are attractive candidates for vaccine target selection as they are evolutionarily robust. Nonamer positions of index incidences ≥ 99% in avian viruses and those completely conserved in human viruses (index incidence of 100%) were selected. Such sequences that overlapped at least one amino acid or were immediately adjacent to each other (not separated by one or more amino acid position) were concatenated. The index sequence incidence criteria of ≥ 99% in avian was chosen because no sequences of at least nine amino acid length were shared between the two viral hosts at 100% incidence. It should be noted that within the concatenated sequences, the starting amino acid of the joined index nonamer sequences are shown in small case. An implication of this is that not all amino acids of the concatenated sequence may meet the incidence criteria set for the index nonamer sequence. Index sequences that were common to viruses of both hosts were further selected for assessment of immunogenicity by searching against reported human T-cell epitopes in the Immune Epitope Database (IEDB) (Vita et al., 2018). Such sequences with nine or more amino acid matches to reported epitopes, with positive T-cell assay results in human were obtained and recommended as vaccine target candidates.

## Results

### Avian and human influenza A (H5N1) protein sequences

Comprehensive collections of protein sequence data were retrieved from the public repository Influenza Research Database for the analyses. The collected and processed H5N1 IAV protein sequence datasets comprised a total of 13,145 full-length and partial sequences for the 12 proteins, with 11,326 avian and 1,819 human H5N1 sequences (Table 1). The six-fold lower number of human H5N1 protein sequences reflects the shorter history and incomplete adaptation of the human virus (Finkelstein et al., 2007). The earliest avian and human H5N1 sequences reported in the database were from 1959 and 1997, respectively, and the viruses originated from a diverse range of geographical areas (Table S1).

**Table 1:** H5N1 avian and human sequences analysed. Data were retrieved from Influenza Research Database (http://www.fludb.org/).

Protein	Avian H5N1			Human H5N1		
	Total sequence collected^a^	Non-redundant^b^	Total sequence analysed^c^	Total sequence collected^a^	Non-redundant^b^	Total sequence analysed^c^
PB1	1845	1026	1026	245	159	159
PB2	1858	1235	1235	249	186	186
PB1-F2	833	250	250	152	52	52
PA	1822	1201	1201	256	256	256
PA-X	1598	725	725	240	118	118
NA	2689	1590	1590	381	249	249
NP	1860	709	709	265	130	130
HA	3987	2410	2410	417	308	308
M1	2015	352	341	264	67	67
M2	1757	427	427	261	83	81
NS1	2072	975	968	251	134	134
NS2	1791	447	444	243	80	79
TOTAL	24127	11347	11326	3224	1822	1819

**Notes.**

a Number of protein sequences collected before removing duplicate sequences.

b Number of protein sequences after removing duplicate sequences (nr dataset).

c Number of protein sequences analysed after alignment quality check, which includes possible removal of irrelevant sequence.

### Dissecting the proteome sequence diversity: scope of the analysis

Shannon’s entropy was used as a measure to quantify the diversity, considering the total number of distinct sequences and their relative probabilities at each aligned overlapping nonamer (nine amino acids) position (1–9, 2–10, etc.) of the proteome. Entropy provided an overview of the diversity, which was then characterized for its components that made up the diversity. All sequences at each of the nonamer positions in the protein alignments were analyzed and classified as characteristic diversity motifs based on the incidences of distinct sequences present at the position, as described in Hu et al. (2013) (Fig. S1). The sequence with the highest incidence (most common) is referred to as the “index” nonamer, while the others are grouped as variants (referred to as total variants), defined as sequences that are at least one amino acid different from the index nonamer. The “major” variant is the second highest incidence nonamer at the position, and thus, the most common variant, while “minor” variants are sequences that occur more than once in the aligned viruses, but each with an incidence less than or occasionally equal to the major variant. “Unique” variants are singletons, each observed only once in the alignment. Additionally, the incidence of all distinct variant nonamer sequences (nonatypes) at a given nonamer position was determined. These diversity motifs were then analyzed to quantify the dynamics of H5N1 IAV proteome sequence change.

### Avian and human H5N1 IAV proteome diversity

**Figure 1:**
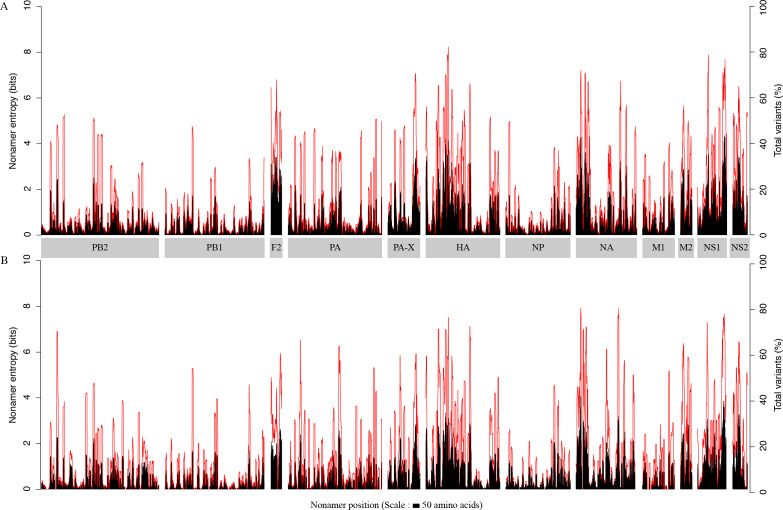
H5N1 entropy and incidence of total variants for each aligned nonamer positions of H5N1 avian (A) and human (B) viral sequences. Entropy (black) and incidence of total variants (red) were measured for each aligned nonamer (nine amino acids) position (1–9, 2–10, etc.) of the proteins. The entropy values indicate the level of variability at the corresponding nonamer positions, with zero representing completely conserved positions (0% total variants incidence) and entropies of ∼4.4 and ∼4.1 were observed to be the highest for viruses of avian and human hosts, respectively.

The overall diversity of the aligned overlapping nonamer sequences for each protein of avian and human H5N1 IAV was measured by use of Shannon’s entropy (Fig. 1). The avian virus proteome had a low average (mean) individual protein entropy, with values ranging from ∼0.4 (PB1) to ∼2.3 (PB1-F2), and a proteome-wide average of ∼0.8. Correspondingly, the percentage of conservation (index incidence) was generally high, with an average individual protein total variants incidence (cumulative of major variant, minor variants and unique variants) ranging from as low as ∼6% (PB1) to ∼39% (PB1-F2), and a proteome-wide average of ∼14%. Half of the proteins had an average entropy value of less than one. The highest absolute entropy value was ∼4.4 for the NS1 protein at position 205–213, with a total variants incidence of ∼73%. However, the highest absolute total variants incidence (∼82%) was for the HA protein at position 170–178, with an entropy of ∼4.1.

The viral proteome in the human host also exhibited a low average individual protein entropy, with values ranging from ∼0.4 (PB1) to ∼1.5 (PB1-F2), and a proteome-wide average of ∼0.7. Correspondingly, akin to the avian viral proteome, the percentage of conservation was generally high, with average individual protein total variants ranging from as low as ∼6% (PB1) to ∼31% (PB1-F2), and a proteome-wide average of ∼14%. However, compared to the avian viral proteome, more than half (∼67%) of the proteins had an average entropy value of less than one. Even in the human viral proteome, PB1 was the most conserved viral protein, with an average entropy of ∼0.4 and an average total variants incidence of ∼6%. There were as many as 320 nonamer positions that were completely conserved amongst the human viruses, versus three for the avian viruses, all of which were in PB1. The maximum absolute entropy for viruses of the human host was ∼4.1 in the NA protein at position 38-46, which also happened to exhibit the highest absolute total variants (∼79%).

**Figure 2:**
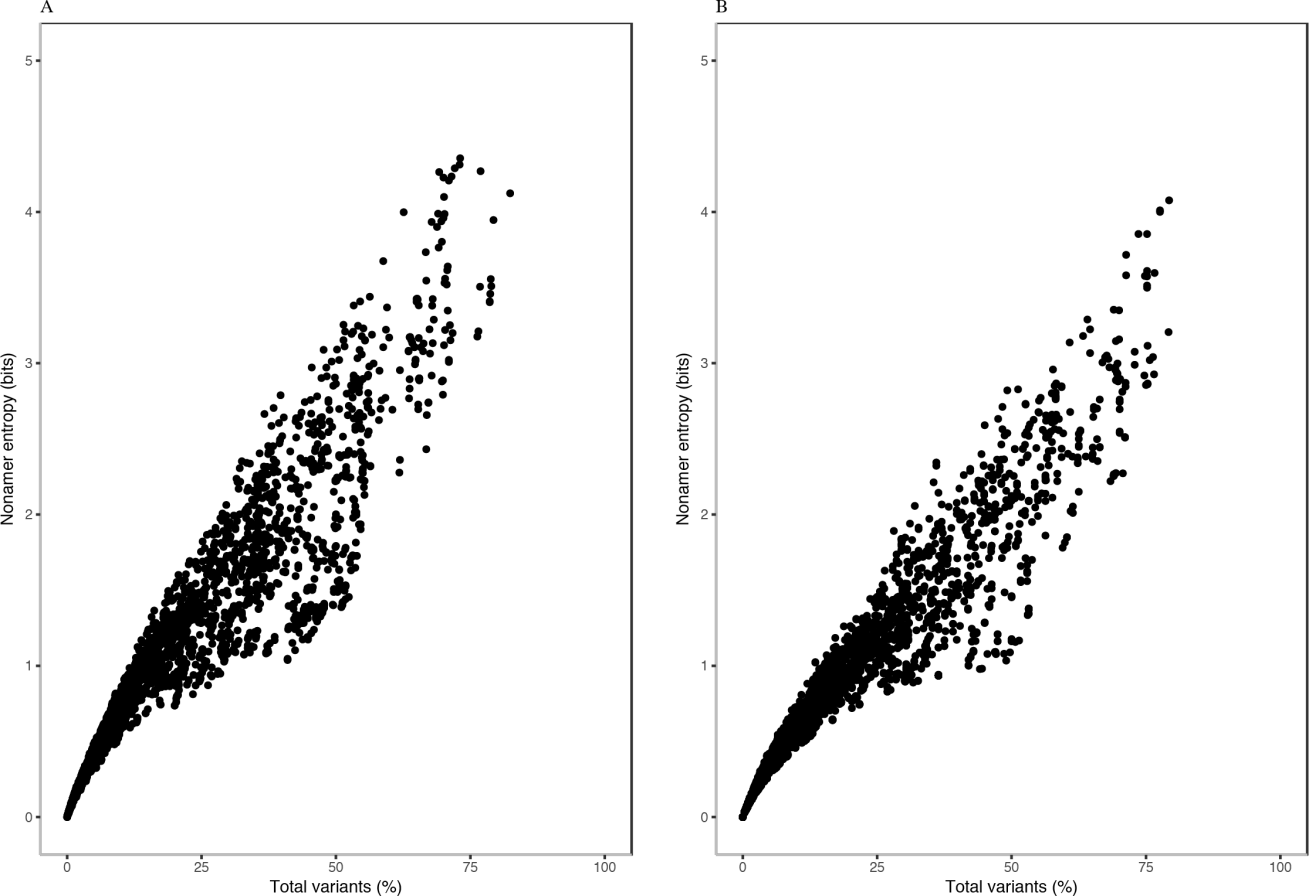
Relationship between entropy and incidence of total variants for (A) avian and (B) human H5N1 influenza A virus proteome nonamer positions. The entropy value for each aligned nonamer position of the avian and human viral proteomes were plotted against the corresponding total variants incidence. A positive correlation was observed for viruses of each host, with a distribution pattern similar between the two. The plot does not show the density of data points.

Each of the H5N1 IAV proteins was found to contain at least a position with a total variants incidence of more than 40%, and a few proteins were with a total variants incidence of more than 60% (Fig. 1). The entropy and total variants showed a positive correlation and a similar pattern between avian and human viruses (Fig. 2). Entropy was zero at all positions that were completely conserved, characterized by the presence of only one distinct sequence at the position (0% total variants). As total variants increased, the range of possible entropy values describing the diversity also increased, with an inflection at approximately 50% total variants, where the entropy range started decreasing, albeit with an increasing value. Notably, there were no positions with total variants higher than 83% in avian viruses and 80% in human viruses, respectively.

Entropy of one was achieved at total variants as low as ∼12% for avian viruses and ∼14% for human viruses. Entropy values of 2.0, 3.0, 4.0 and the maximum of ∼4.4 were achieved for avian viruses at total variants of ∼29%, ∼46%, ∼63% and ∼73%, respectively. Likewise, it was at ∼35% (entropy of 2.0), ∼58% (entropy of 3.0), ∼78% (entropy of 4.0), and ∼79% (maximum entropy of ∼4.1) total variants for human viruses.

### Quantitative analysis of avian and human H5N1 IAV: Characterization of diversity motifs

The protein sequence diversity of H5N1 IAV was further examined by quantitative analysis of characteristic diversity motifs at each of the aligned nonamer positions of the two viral proteomes. There were 19 nonamer-position deletions in human viruses compared to one amongst avian viruses) (Tables S2; S3). An example of the quantitative analysis is shown with 13 aligned nonamer positions of HA (158–178) (Table 2). The dataset for each of the 13 aligned nonamer positions contained 2,349 to 2,353 sequences. The first position, HA (158-166) was the most conserved with an entropy of ∼0.6, and index nonamer incidence of ∼92%. The remaining, small fraction (∼8%) of the aligned sequences at this position consisted of a major variant (∼5%), minor variants (∼2%), and unique variants (<1%). The incidence of the distinct variant nonamer sequences (nonatypes) at this position was approximately 1%, represented by the single major variant and different minor and unique variants. In contrast, the last position, HA (170–178), of the selected 13 nonamer positions was highly diverse, with a high entropy of ∼4.1 and a low index incidence of ∼18%. The total variants (∼82%) of the aligned nonamers at this position comprised of ∼14% major, ∼66% minor, and ∼2% unique, with a nonatypes incidence of ∼4%. The remaining HA nonamer positions (159–177) were of mixed-variability, with index incidences of between 20–90%.

**Table 2:** A sample of the quantitative diversity analysis for HA protein of avian Influenza A (H5N1) subtype. The full data is available in Table S2.^1^

Protein	Aligned nonamers			H(x)^c^	Index^d^		Variants^e^				Nonatypes^i^
		Position^a^	No.^b^		Sequence	[%]	Total	Major^f^	Minor^g^	Unique^h^	
							%				
HA	#	158–166	2353	0.6	SFFRNVVWL	92	8	5	2	<1	1
	&	159–167	2352	1.1	FFRNVVWLI	83	17	8	9	1	1
	&	160–168	2353	1.1	FRNVVWLIK	83	17	7	9	1	1
	&	161–169	2352	0.9	RNVVWLIKK	88	12	7	4	1	1
	&	162–170	2351	1.7	NVVWLIKKN	64	36	23	12	1	2
	&	163–171	2351	2.8	VVWLIKKNS	30	70	26	43	1	2
	&	164–172	2350	3.5	VWLIKKDNA	21	79	20	57	1	3
	&	165–173	2350	3.4	WLIKKDNAY	21	79	21	57	1	3
	&	166–174	2350	3.4	LIKKDNAYP	21	79	21	57	1	3
	&	167–175	2349	3.5	IKKDNAYPT	21	79	21	57	2	4
	&	168–176	2349	3.2	KKDNAYPTI	24	76	21	54	1	3
	&	169–177	2349	3.2	KDNAYPTIK	24	77	21	54	1	3
	^+^	170–178	2350	4.1	NSTYPTIKR	18	82	14	66	2	4

**Notes.**

1 All percentages are shown to the nearest whole number.

a Amino acid number at the start and end of the nonamer position in the protein alignment. The symbol # denotes a highly conserved nonamer position (index incidence ≥ 90%), & denotes a mixed-variable position (index incidence between 90% & 20%), and + denotes a highly diverse nonamer position (index incidence ≤ 20%). See Fig. S1 for the definition of diversity motifs.

b Total number of protein sequences analysed at the aligned nonamer position; the difference in number between the nonamer positions was due to the inclusion of both partial and full-length sequences in the alignment.

c Shannon’s nonamer entropy, which indicates the level of diversity of the nonamer sequences at the position (H(x); see Fig. 1 for details).

d The index nonamer is the most prevalent sequence at the position.

e Variants are nonamer sequences that differ by one or more amino acids from the index sequence.

f The major variant is the second most common sequence at the position.

g Minor variants are multiple different repeated nonamer sequences, each occurring more than once and with an incidence of less than or occasionally equal to the major variant.

h Unique variants are nonamer sequences that are observed only once at the position.

i Nonatypes are distinct sequences among the variants for a given position; for example, the position 170–178 had 103 distinct nonamer sequences (Data S1; from a total of 2,350 sequences), therefore the percentage of nonatypes for this position is ∼4.4% ((103/2,350) ×100%).

### Dynamics of protein sequence diversity

**Figure 3:**
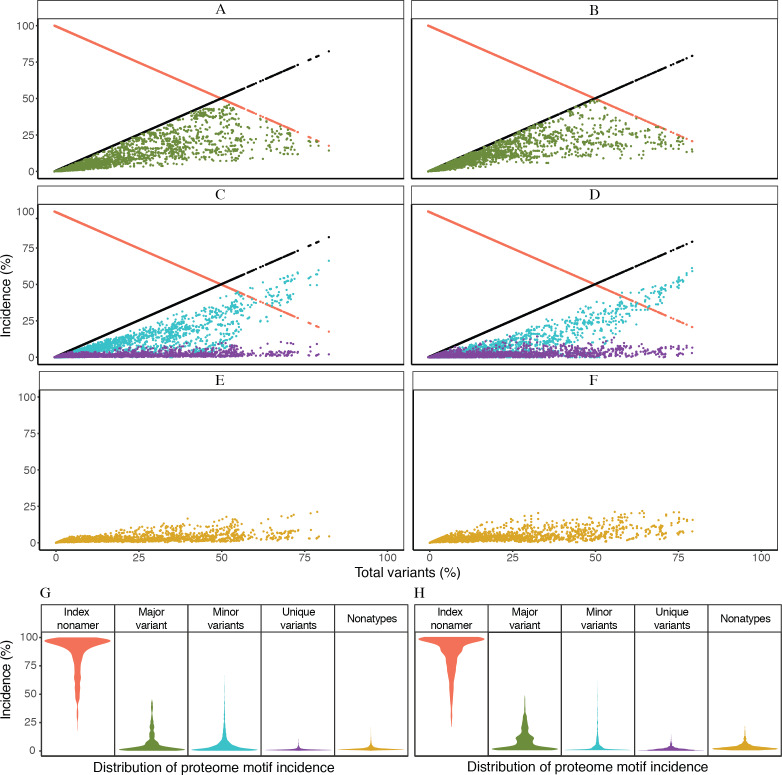
Dynamics of diversity motifs for avian (A, C, E, G) and human (B. D, F, H) H5N1 influenza A virus proteomes. (A–F) Motif incidence (index, major, minor, unique, and nonatypes) in relation to increased total variants incidence. (G–H) Violin plot depicting the frequency distribution of the incidences for the various motifs. The colours for panels A–F follow the labels in panels G–H. The width of the plot (*x*-axis) represents the frequency distribution of a given incidence of the indicated motif. The wider the plot, the higher the frequency.

Based on the definitions of the diversity motifs, each individual variant motif had a distinctive pattern of incidence in relation to increased total variants (Fig. 3). These patterns were generally similar between the avian and human viruses (Figs. 3A–3F). The major variant, the second most prevalent sequence of the aligned population, was transient with a pyramidal peak incidence of ∼46% for avian viruses and ∼49% for human viruses. The multiple different minor variants, each with an incidence lower or occasionally equal to that of the major variant, represented the principal variant motif, particularly for positions with total mutations (variants) of ≥ 50%. The unique variants were enigmatic as they were present for nearly all of the positions with non-zero total variants, albeit with a low proteome-wide average incidence of ∼1% for viruses of both avian and human hosts (maximum incidence of ∼11% and ∼14%, respectively). Nonatypes, a measure of multiple distinct variant sequences, occurred at nearly all of the positions (∼99% and ∼93% for viruses of avian and human, respectively), with a proteome-wide average incidence of ∼2% for distinct variants amongst avian viruses and ∼3% amongst human viruses. Notably, highly diverse nonamer positions (total variants of ≥ 80%) were almost non-existent in the H5N1 IAV proteomes of both avian and human viruses (one position and none, respectively).

The distinctive character of the dynamics of the individual variant motifs for the H5N1 IAV proteomes from avian and human hosts is demonstrated by the relationship between motif concentration and increased mutations (Figs. 3A–3F). The decrease in conservation, represented by decline of index sequence incidences from 100% to ∼18%, was inversely proportional to the increase in total variants incidences, 0% to ∼82%. This inverse relationship appeared to be in a continuum for positions with total variants of less than 50%, but became sparsely distributed closer to the maximum observed total variants.

Each of the three variant motifs (major, minor, and unique) displayed a characteristic pattern of incidence change in relation to increased total variants. The major variant, which by definition does not exceed the incidence of the index sequence, exhibited a restrictive pyramidal incidence pattern. As such, the major variant can have a maximum incidence at 50% total variants, limited by the incidence of the index. Prior to the peak incidence, the major variant increases in incidence with increased total variants and reducing index incidence. However, a reverse is observed for the major variant after the peak incidence; its incidence starts decreasing with the continued decrease in index incidence.

The minor variants, defined as sequences less prevalent than the major variant but occurred more than once, were collectively the predominant variant motif for majority of the positions with index incidence of <50%. Unique variants comprised the remaining population of variant sequences. They represent viral strains observed only once in the recorded virus population. They were characteristically distinctive for being present at nearly all of the positions with non-zero total variants, including the highly conserved positions. The incidence of unique variants progressively increased, with a maximum incidence of ∼11–14% for viruses of avian and human hosts, respectively, as total variants inceased; this was despite the presence of other variant motifs. The fact that the unique variants were present at nearly all of the nonamer positions, including highly conserved, of every protein (Tables S2 and S3), indicates that they are not artifacts of sequencing or premature sequences. Nonatypes, a measure of different variant sequences at a given position, were generally of low incidence (proteome-wide average: ∼2% and ∼3%) with the highest incidence of ∼21% and ∼22% at positions corresponding to total variants of ∼79% and ∼64% (avian and human viruses, respectively), which principally comprised of different minor and unique variants.

Frequency distribution of the diversity motifs incidences for H5N1 IAV proteomes of avian and human viruses were studied by the use of violin plots (Figs. 3G–3H). Overall, the index nonamer was the principal motif, with a proteome-wide average incidence of about 86%. The proteome-wide, predominant mutation of the index was the major variant, with a greater incidence than any of the other variant motifs for ∼51% and ∼77% of the avian and human proteome nonamer positions, respectively. All the variant motifs (major, minor, unique) were predominantly of low (<10%) incidence, as illustrated in Fig. 3, with the exception of minor variants showing a collective increase in incidence, even surpassing the index, at positions of more than 50% total variants. Nonetheless, the fraction of positions showing this increasing trend of minor variants incidence greater than that of the index was minimal (<2%) for both avian and human viruses. The nonatypes, which typically would comprise of a single major variant, multiple different minor variants, and all the unique variants, were also mainly of low incidence (proteome-wide average: ∼2–3%), similar to the incidences of unique variants (proteome-wide average of ∼1%).

**Figure 4:**
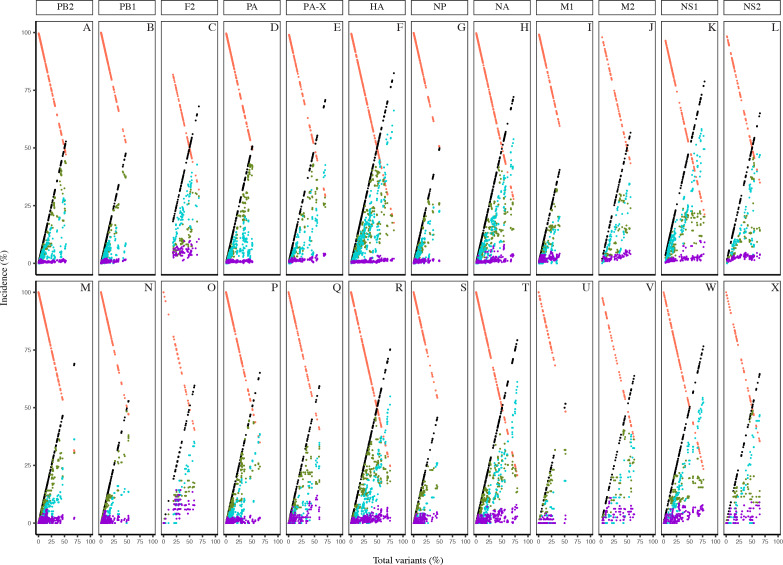
Dynamics of diversity motifs for avian (A–L) and human (M–X) H5N1 influenza A virus proteins. Motif incidence (index, major, minor, unique, and nonatypes) in relation to increased total variants incidence at the protein level.

**Figure 5:**
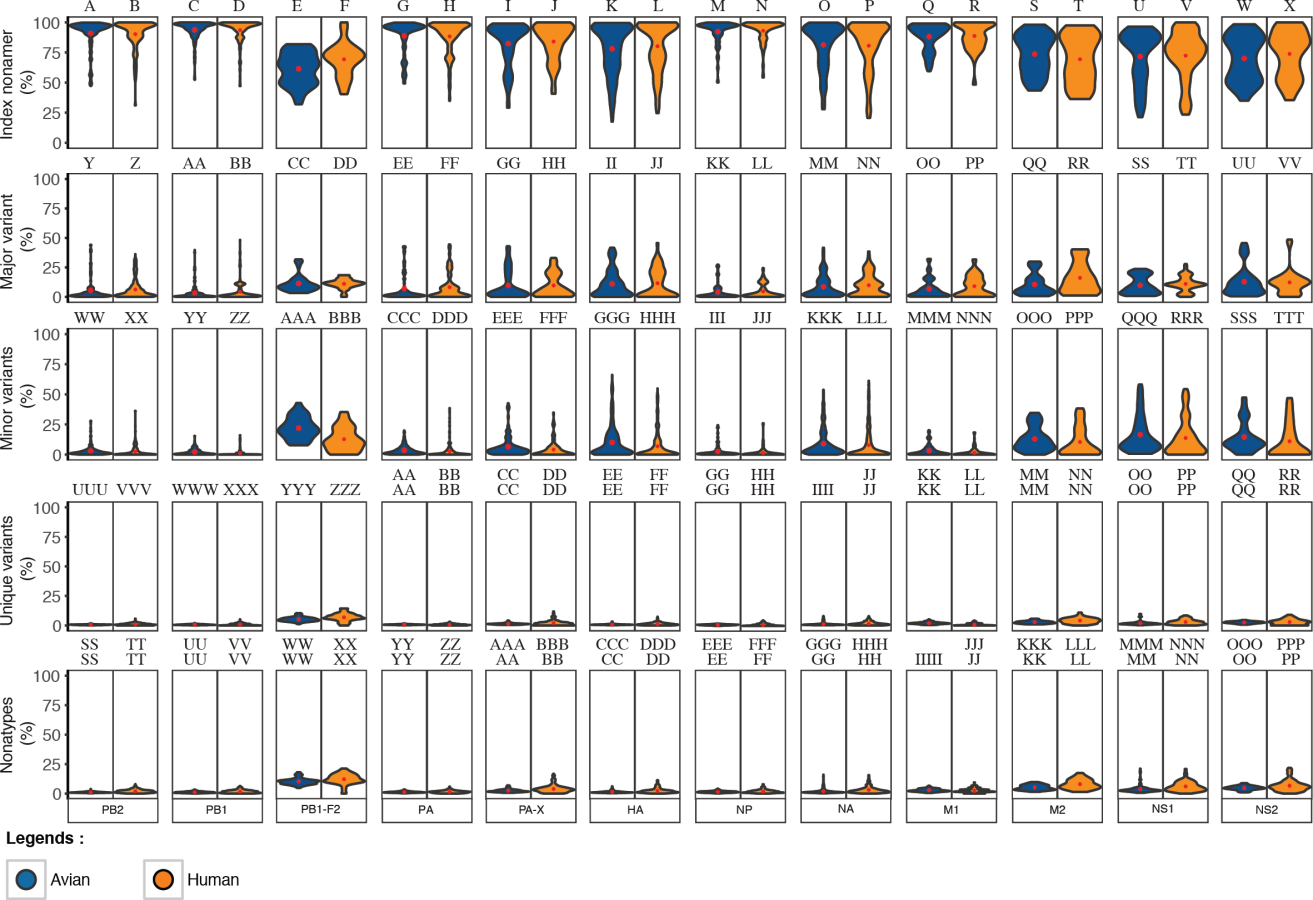
Frequency distribution violin plots of the diversity motifs for H5N1 influenza A virus proteins. Violin plot depicting the frequency distribution of the incidences for the various sequence motifs at the protein level. The width of the plot (*x*-axis) represents the frequency distribution of a given incidence of the indicated motif. The wider the plot, the higher the frequency. The red “x” mark represents the mean incidence value.

Collectively, the dynamics of the diversity motifs for the individual proteins were generally in agreement with the proteome patterns for both the avian and human viruses (Figs. 4 and 5). This was particularly so for avian viral proteins, HA, NA, PA-X, and NS1, and human viral proteins, HA, NA and NS1; all of which are generally diverse and exhibited at least one position with a total variants incidence of ≥ 70%. However, a number of the avian viral proteins, such as M1 and PB1 did not have any data points for some ranges of total variants, starting from as low as ∼40%. In contrast, the relatively diverse protein, avian PB1-F2 did not have any data points for total variants below ∼18%. Additionally, there was a distinction in the dynamics of the diversity motifs for majority of the individual proteins between avian and human viruses, such as, in particular PA, PB1-F2, and M1.

### Motif switching

**Figure 6:**
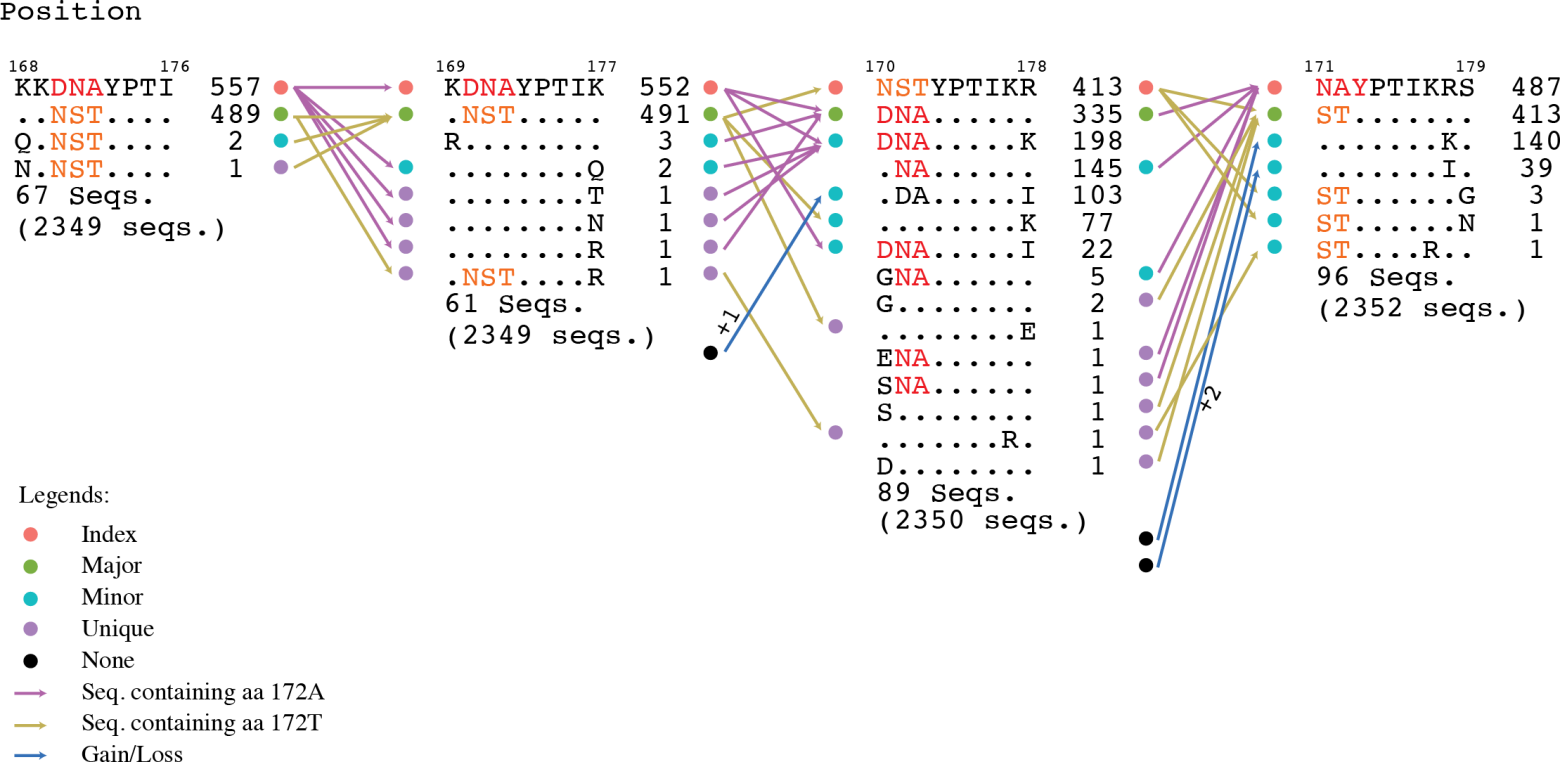
Motif switching. An example of motif switching events observed in avian HA protein. The alignment region shown is from position 168 to 179, comprising of four overlapping nonamer positions. At the nonamer position 168–176 (with 2,349 protein sequences aligned at the position), the nonatype KKDNAYPTI containing the amino acid (aa) “A” at position 172 (172A) is the predominant sequence, and thus, the index, while the nonatype KKNSTYPTI containing the aa “T” at the corresponding position (172T) is the second most predominant sequence, and thus, the major variant. The nonamer position also included multiple nonatypes of minor and unique variants (two nonatypes shown: a minor and a unique variant; the remaining 67 nonatypes not shown). In the subsequent nonamer position (169–177), the nonamer containing aa 172A remained as the index, but with a reduced incidence because a small fraction (five sequences) of the index (from the preceding position 168–176) split into four different nonatypes because of a variant amino acid in each, as a result of the position shift, which contributed to the occurrence of one new minor and three new unique variants at the current focal nonamer position (169–177). Meanwhile, the nonamer containing aa 172T, which was the major variant at the preceding position, remained as major variant at the current focal nonamer position, but with an increase in its incidence because two nonatypes (a minor variant of two copies and a unique variant) from the preceding nonamer position (168–176) merged with the major variant at the current focal position 169–177. Nonetheless, the major variant of the preceding position also underwent a split, where one copy became a new unique variant at the current focal position. In the next nonamer position (170–178), an index switching event is observed, where the nonamer containing aa 172A has significantly dropped in incidence to be downgraded from the previous index rank to now as a major variant, and replaced by the nonamer containing aa 172T (previously the major variant) as the new index. As many as 217 copies (552 − 335;∼39%) of the former index sequence were split into different variant motifs, thus decreasing the incidence of the sequence. Although the nonamer containing aa 172T also split into other variants, the incidence drop was less (∼16%), leaving it as the sequence with the highest incidence and thus the predominant index. In the subsequent nonamer position (171–179), another index switching event occurred, which witnesses a reversal of the previous switch, where now the nonamer containing aa 172A is back as the index and the nonamer containing aa 172T is back as the major variant. This was due to the merger of a significant copy number of different minor and unique variants with the major variant (nonamer containing aa 172A), and thus, resulting in an increase in its incidence at the current focal position 171–179 and becoming the predominant index. At the current focal position, it was also observed that variants can be gained from a motif classified as “None” (due to indel) at the preceding position. Similarly variants can become loss, classified as “None” if the position shift introduced an indel.

Motif switching, another relevant finding, revealed that there were a significant number of nonamer positions in the proteome where fitness change of one or more amino acids, such as through mutations, changed the incidence of a given nonamer sequence across its overlapping positions, resulting in a sequence rank change, and thus, a motif change. Although, this was observed for all the motifs (index, major, minor or unique), index switching was noteworthy, where a major variant replaced the prevalent index sequence. An example illustrating motif change is provided in Fig. 6.

Motif switching appeared to be common across the avian viral proteome as exemplified by both avian PB2 and PA-X proteins (∼88% and ∼89% of the protein nonamer positions). The occurrences of motif switching in these two proteins for the human viral proteome were also common, but relatively less (∼50% and ∼53% of the positions). Although the data studied was for PB2 and PA-X, we expect a similar observation for all the other proteins.

Index switching, though noteworthy, was not omnipresent; it was only observed at three positions of the avian viral proteome (two in HA and one in PA-X) and six positions of the human viral proteome (one in HA, four in NA, and one in PB1). Switching between the other motifs were more common.

Nonamer positions that exhibited index switching were observed to be generally non-immune relevant (Table S4). Six of the eight positions were predicted to be non-immune relevant for both the index and the major variant; and thus, no epitope gain. One position had both the index (94 YIVEKINPA 102 in HA of the human viral proteome) and the major variant (YIVEKINPV) predicted to be HLA class I supertype-restricted epitope (A2 and A26 for each); and thus, no epitope loss. The other one position (214 KKSYLIRAL 222 in PB1 of the human viral proteome) only had the major variant (KRSYLIRAL) predicted as a supertype-restricted epitope (B27 and B39), with the dominant index as non-epitope (epitope loss). Thus, in summary, seven positions showed no change between the preceding and the index switching position; with only one exhibiting a putative T-cell epitope loss. Similarly, none of the eight positions were predicted to be immune-relevant for HLA Class II DR supertype and linear B-cell epitope; and thus, no change for all the positions. On a separate note, of the eight index switching positions, only one was highly conserved with a ∼82% intra-subtype (H5N1) incidence, and the remaining were of higher variability with intra-subtype (H5N1) incidence of below 55%, to as low as ∼18% (Table S4).

### Distribution of conserved and variable nonamer positions in avian and human H5N1 IAV proteomes

**Figure 7:**
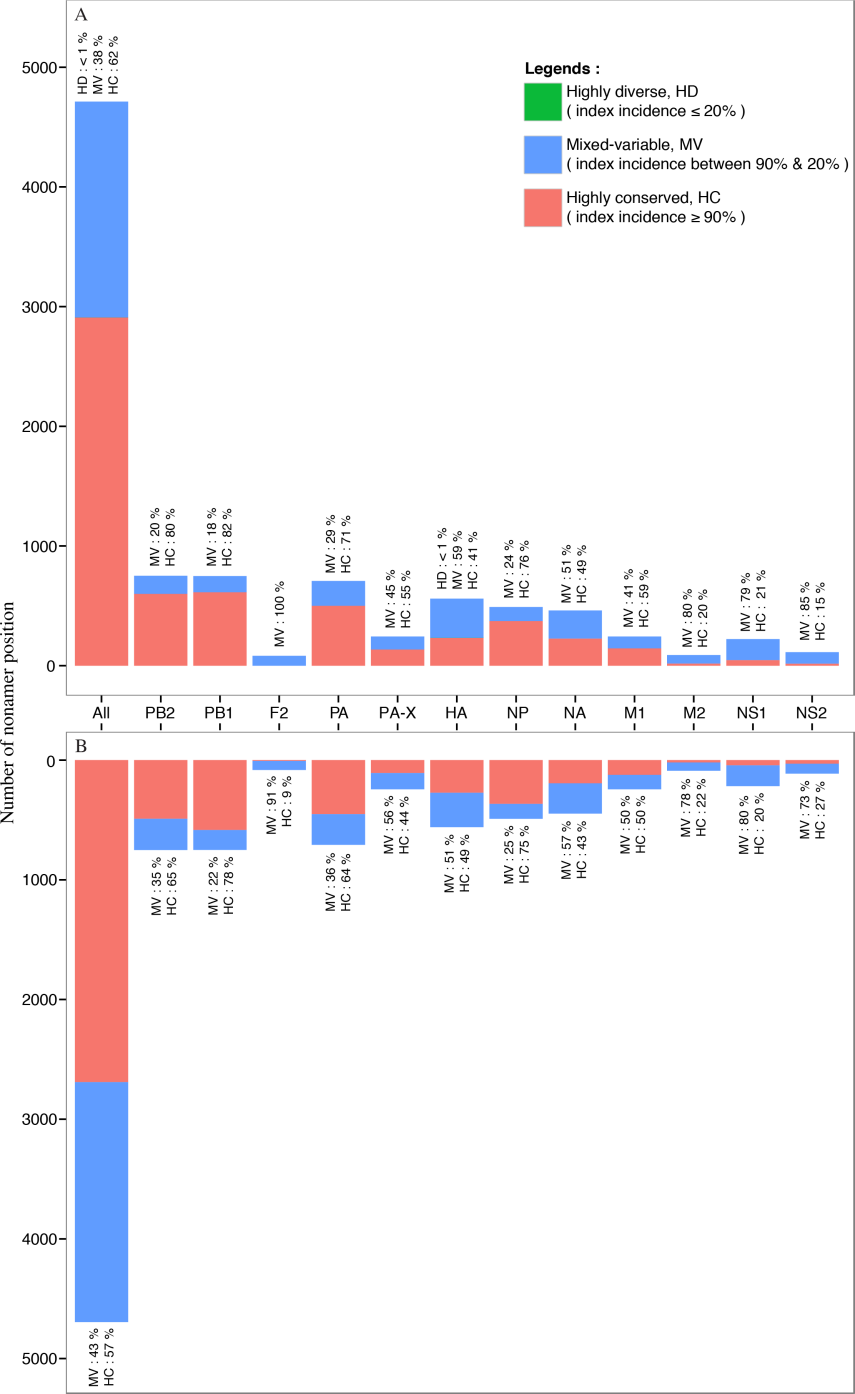
Distribution of conservation level of nonamer positions. The nonamer positions of the proteome and the individual proteins were defined as highly conserved (red, index incidence ≥ 90%), mixed-variable (blue, index incidence < 90% & > 20%), and highly diverse (green, index incidence ≤ 20%). Majority of the nonamer positions were highly conserved, representing ∼62% and ∼57% of avian (A) and human (B) H5N1 proteomes, respectively. Only one position was identified as highly diverse, which represented less than 1% (avian, HA protein), while mixed-variable positions represented ∼38% of the avian H5N1 proteome and ∼43% of the human H5N1 proteome. The M1, NP, PA, PB1 and PB2 proteins were observed to have a higher percentage of highly conserved positions in both avian and human H5N1 proteomes.

Nearly all of the nonamer positions of the avian and human H5N1 IAV proteomes were classified as highly conserved (index incidence ≥ 90%) or mixed-variable (index incidence between 90% and 20%) (Fig. 7). Highly conserved positions, defined as those with index sequence incidence of 90% or more among the aligned viruses, represented ∼62% and ∼57% of the avian and human H5N1 IAV proteomes, respectively. Seven percent (∼7%; 320 positions) of the human H5N1 IAV proteome was completely conserved (index of 100% incidence), and 264 of these 320 can be considered evolutionarily conserved given the relatively larger sample size (≥100 sequences) in the alignment. In contrast, a smaller fraction (<1%; 3 positions) of the avian viral proteome was completely conserved, observed only in the PB1. Given that less than 7% of the human and avian viral proteomes were completely conserved, nearly all nonamer positions of the aligned viral proteins of both proteomes contained variants with one or more mutations to the index sequence. Notably, only one position (170–178 of HA) in the avian H5N1 IAV proteome was classified as highly diverse (index incidence ≤ 20%) with total variants of ∼82%, and there were none in the human viral proteome.

Randomly selected nonamer positions corresponding to H5N1 IAV of both avian and human hosts were analyzed for effects of sequence change to T-cell epitopes at the three conservation levels (Table S5). Number of nonatypes for the avian viruses ranged from eight to 104 at these positions (highly conserved: three (3); mixed-variable: three (3); and highly diverse: only one such position). Nonatypes for the human viruses ranged from eight to 24. Index sequences were shared between viruses of the two viral hosts for all the positions studied. Similarly, for all these positions, majority of the nonatypes for the human viruses were shared with the avian viruses. The shared sequences were generally non-epitopic. The human H5N1 IAV non-shared sequences were generally also non-epitopic. The avian H5N1 IAV non-shared sequences were generally also non-epitopic, sans for one mixed-variable position 497-505.

### Identification of highly conserved, immunogenic sequences as potential vaccine targets

A total of 302 and 320 nonamer index sequences were identified to meet the conservation criteria of ≥ 99% incidence and 100% incidence for avian and human viruses, respectively. Concatenation of these sequences resulted in 52 and 69 highly conserved, distinct sequences for avian and human H5N1 viruses, respectively. These sequences were found in each of the viral proteins of both hosts, except in M2. Twenty-five (25) of these sequences were shared between viruses of the two hosts, with a match of at least nine consecutive amino acids. Twelve (12) of these shared sequences were found to match at least nine consecutive amino acids of 30 reported human T-cell epitopes in IEDB (Table 3), many of which were restricted to multiple HLA alleles, thus relevant to a larger proportion of the human population. Additionally, 12 of the 25 shared sequences were pan-subtype conserved as they matched at least nine consecutive amino acids of sequences reported by Heiny et al. (2007) that were conserved across H1N1, H3N2, and H1N2 subtypes (≥80% incidence in each subtype). Eight (8) of these 12 were also those that matched the reported T-cell epitopes (Table 3).

**Table 3:** Reported human T-cell epitopes in the H5N1 highly conserved sequences.

Protein	**H5N1 highly conserved sequence**^ a^	**Immunogenic T-cell epitope**^b^			
		**EpitopeID**^c^	**Sequence**^d^	**Tsubset**	**HLA Allele**
M1	175 ** HENRMVLAS** 183	65389	TNPLIR**HENRMVLAS**TTAKA	CD4+	HLA-DRB1*01:03, HLA-DRB1*15:01, HLA-DRB5*01:01, HLA-DRB1*07:01, HLA-DRB1*11:01, HLA-DRB1*15:02, HLA-DRB1*03:01
		148697	NPLIR**HENRMVLAS**T	CD4+	HLA-DR
		79948	NPLIR**HENRMVLAS**	CD4+	HLA DRA*01:01/DRB1*07:01
		113695	PLIR**HENRMVLAS**T	CD4+	HLA-DRB1*03:01
		97403	IR**HENRMVLAS**TTAKAM	CD4+	–
		97395	IK**HENRMVLAS**TTAKAM	CD4+	–
NP	68 ** LSAFDERRN** 76	54595	RLIQNSLTIERMV**LSAFDERRN**K	–	–
		97689	TIEKMV**LSAFDERRN**KYL	–	–
		54955	RMV**LSAFDERRNR**YLEEHPS	CD4+	HLA-DRB1*01:01
		164364	RMV**LSAFDERRN**KYLEEH	CD4+	–
	137 ** MIWHSNLND** 145	41793	**MIWHSNLNDA**TYQRTRALVR	CD4+	HLA-DRB1*07:01, HLA-DRB5*01:01
	218 **AYERMCNILKGK** 229	18366	FWRGENGRKTRS**AYERMCNILKGK**	CD4+	HLA-DR1, HLA-DR2
		97621	RRTRI**AYERMCNILKGK**F	CD4+	–
		97607	RKTRS**AYERMCNILKGK**F	CD4+	–
		145824	I**AYERMCNILKGK**FQTAA	CD8+	HLA-B*15:01
		181251	**YERMCNILKG**	CD8+	HLA-B44, HLA-B*18:01
	271 **AHKSCLPACVYG** 282	4255	ARSALILRGSV**AHKSCLPACVYG**P	–	–
		27284	ILRGSV**AHKSCLPACVYG**LA	CD4+	HLA-DRB1*07:01
		33285	**KSCLPACVY**	CD8+	HLA-A*01:01, HLA-A*30:02
PA	583 **RCLLQSLQQ** 591	129181	KWGMEMR**RCLLQSLQQ**I	–	–
PB1	26 ** GDPPYSHGTGTGY** 38	97682	TFPYT**GDPPYSHGTGTGY**	CD8+	–
		75755	**YSHGTGTGY**	CD8+	HLA-A*01:01, HLA-A*26:01HLA-A*30:02, HLA-A*29:02
	337 ** LSIAPIMFS** 345	236661	YITRNQPEWFRNV**LSIAPIMFS** NKMARLGKGYMFE	–	–
		483463	QPEWFRNV**LSIAPIMFS**NK	–	HLA-DR
		148616	FRNV**LSIAPIMFS**NKM	CD4+	HLA-DR
	401 **ASLSPGMMMGMFNMLSt** 417	42143	**MMMGMFNML**	–	HLA-A2
	500 ** GFVANFSMELPS** 511	97627	RY**GFVANFSMELPS**FGV	CD8+	–
		97314	**FVANFSMEL**	–	HLA-A2, HLA-A*02:01
	537 ** NDLGPATaqMAlqlfiK** 553	21574	**GPATAQMAL**	CD8+	HLA-B7
	555 **YRYTYRCHRGD** 565	1960900	D**YRYTYRCHRGD**TQIQT	CD4+	HLA-DRB1*03:01

**Notes.**

a Amino acids are numbered according to the sequence alignment of the respective protein. Positions that do not satisfy the concatenating criteria are indicated as small letter case. Sequences that matched with Heiny et al. (2007) data are underlined.

b Reported T-cell epitopes from the Immune Epitope Database (IEDB). Dashes, not determined.

c Epitope ID based on IEDB (http://www.iedb.org/).

d Immunogenic sequences in IEDB that matched the highly conserved sequences are bold in red.

## Discussion

In this study, we applied a bioinformatics approach to characterize the diversity of avian and human H5N1 IAV protein sequences as different viral variant motif populations and illustrate the dynamic interplay between them. The publicly available protein sequence data, with reported viral isolate date range of at least 53 years (1959-2012), provides a compendium of the known sequence variants of H5N1 IAV. A similar study for HIV-1 clade B viruses, evaluating the amino acid make-up and incidence of the distinct sequence changes, in the context of antigenic diversity, had been reported by Hu et al. (2013).

Mutations of influenza and other viruses are manifsted in the interrelated dynamics of the proteome-wide distribution and the population-wide incidence of the sequence change. The index sequence, with an incidence range of 100% to ∼18%, had an inverse, linear relationship to the total concentration of mutations (total variants: 0% to ∼82%). Notably, highly diverse (index incidence ≤ 20%) positions were non-existent, except for the single nonamer position HA 170-178 of the avian viruses. This suggests a functional constraint (Saakian et al., 2006) preventing the virus population from exploring a hypothetical peak fitness in the highly diverse region. This is in contrast to HIV-1 clade B viruses, which exhibited a large fraction of positions within the highly diverse range; such positions are thought to contribute to the general collapse of the early immunity to the virus (Hu et al., 2013). In keeping with the quasispecies model, the progeny viruses of H5N1, thus, do not seem to be capable of sequence plasticity as observed for certain positions of the HIV-1 genome (Hu et al., 2013); perhaps this restriction for H5N1 is imposed by the need for the virus to survive in a diverse range of host species. Nonetheless, the observed patterns of H5N1 IAV sequence change seem sufficient in providing a spectrum of viral variants that enable the virus population to utilize changes in selection pressure and aid in the long-term evolutionary stability and versatility of the virus within the repertoire of the hosts. This pattern of sequence change supports the notion of a Darwinian dominant replicator (index) and a “clan” of variant molecules that can also have maximum reproductive fitness (Eigen & Schuster, 1977).

The major mutation, which by definition must have an incidence of less than that of the index, is limited to a maximum incidence of 50% and must decrease with decreasing incidence of the index. In general, a large proportion (∼93% for both avian and human viruses) of the major variants had an incidence of <25%, with some of the remaining nearly matching the incidence of the index as total variants increased to more than 50%. The dominance of an index that is an antigenic epitope is challenged by the major variant with increasing total variants, alluding to the possibility of an index switch (Hu et al., 2013), where fitness increase of one or more amino acid mutations may result in a dominance switch between the major variant and the prevailing index.

As there can only be a single major mutation at a variant nonamer position, by definition, therefore, the scope of different sequences of this motif is strictly limited. Thus, sequence diversity of influenza is embodied in minor and unique mutations, which can exist as many different sequences at the variant nonamer positions. This is particularly for positions with more than 50% total variants, where the minor variants, each with an individual incidence less than or equal to the major variant, were collectively the dominant variant population for most of the positions. Notably, for a small fraction of the positions (<2%), the combined incidence of the minor variants was greater than the predominant index. Minor variants are, thus, a highly dynamic variant population of the proteome; they are a product of the interplay between fitness-selection and continued mutation of the index sequences and major variants. The different sequences of the minor variants alone present a spectrum of sequences that could escape immune recognition or act as altered peptide ligands, affecting the recall of pre-existing immunity (Evavold, Sloan-Lancaster & Allen, 1993; Sloan-Lancaster & Allen, 1996; Zinkernagel & Klenerman, 1998; Kim et al., 2009; Pan & Deem, 2012; Park et al., 2016).

Notably, there was no significant change in the incidence of the unique variants at the positions with more than 50% total variants, unlike in the case of HIV-1 clade B viruses (Hu et al., 2013), where it increased dramatically for the hyper-variable positions (≥90% total variants); these positions do not exist for H5N1 IAV. Although the average proteome-wide incidence of the unique variants was about 1%, thus, posing questions on their validity, they were observed at the majority of the proteome nonamer positions and, for some, attained a higher incidence (maximum ∼14%) as total variants increased. Similarly, nonatypes, a measure of distinct variant sequences, was chiefly of low incidence (proteome-wide average: ∼2% and ∼3% for avian and human viruses), with increase in incidence (to a maximum of ∼21% and ∼22%, respectively) nearing positions with total variants of ∼79% and ∼64%, respectively. Such positions, which comprised largely of different minor and unique variants, represent selected hotspots for mutation, with implications for viral immune evasion. Notably, the lack of highly diverse positions, where the difference in incidence between the different motifs, including the index, is almost negligible and that switch of motif prevalence is possible, suggests a relatively dominant role of the index sequences for H5N1 viruses.

Index switching was previously described by Hu et al. (2013)*,* and that it can effect a fitness change. However, we observed that this was uncommon across the avian and human viral proteomes. In contrast, switching between the other motifs was more common and collectively may have a larger role in contributing to the fitness selection of the virus. The motif switching phenomenon, also alluded by Hu et al. (2013), was not only observed for both the proteomes, but was extensive and complex; it is thought to be a contributor to fitness selection in other viruses as well, in particular those of quasispecies nature. The dynamic nature of motif switching can be attributed to merging, splitting, and gain/loss of nonatypes between the nonamer positions. Nonatype merging is observed when two or more distinct nonamers of a given incidence at a position combined/merged to become one in the subsequent position. Conversely, nonatype splitting is when a distinct variant nonamer separates into two or more distinct variant nonamers in the subsequent position. As for nonatype gain, it occurred when a motif was not present in the previous position but appeared in the subsequent position, and *vice versa* for the loss; this was largely due to indels and in some cases because of our filtering of nonamers with ambiguous amino acids. Motif switching was common at all the three conservation levels (highly conserved, mixed-variable, and highly diverse) for both the avian and human viral proteomes. Positions of motif switch are hotspots for sequence change. Among the motif switch, index switching can be disruptive and is of concern for selection of vaccine target candidates. Positions that do not exhibit index switching may be considered as additional criteria for vaccine target selection.

The consequence of index switching to the immune-relevance of the affected nonamers was evaluated. The index and the major variants of the affected positions, generally, did not appear to be immune relevant, for both humoral and cellular responses; only one position demonstrated epitope loss due to index switching. Also, index switching positions (or the preceding positions) appeared unlikely important for preservation of viral function because they were generally of low intra-subtype (H5N1) incidence, below 55%. This suggests that the index switching positions may be of structural in nature, providing fitness benefit to the virus.

Vaccines against influenza have been developed empirically (Siegrist, 2013), comprising of H1N1, H3N2, and influenza B strains that are predicted to be circulating in the subsequent season, and are not inclusive and protective against pandemic potential viruses, such as H5N1 and the most recent H7N9 (Meng et al., 2014). The lengthy process of vaccine strain selection is multifaceted at every stage, and it takes almost four months to select candidate vaccine strains (World Health Organization, 2007). Moreover, sequence variability poses a significant problem in vaccine design against viruses. For example, a study by Park et al. (2004) to assess the effect of strain heterology showed increased risk of disease for heterologously compared to homologously vaccinated animals. Hence, highly conserved sequences are thought as a solution in selecting vaccine targets that are representative of the majority of influenza viruses. Several studies (Assarsson et al., 2008; Cassone & Rappuoli, 2010; Steel et al., 2010; Pica & Palese, 2013; Lee et al., 2014; Deng et al., 2015; Huber et al., 2015) have provided evidence supporting the notion of conserved sequences as universal vaccine targets. Steel et al. (2010) demonstrated that a vaccine based on the highly conserved HA stalk region was broadly protective in a murine influenza model. Other studies (Cassone & Rappuoli, 2010) identified human antibodies that bound to highly conserved epitopes of the HA, NP or the M2 for generation of cross-protective antibodies and cell-mediated immunity. Huber et al. (2015) constructed a peptide-based vaccine of conserved parts (HA, M2, NP, PB1, and M1) of influenza A virus, containing B and T-cell epitopes, which showed promising results in both mice and ferrets. Assarsson et al. (2008) identified 54 conserved IAV epitopes tested against peripheral blood mononuclear cells (PBMC) from 44 healthy human blood donors. A few studies have evaluated conserved sequences of the M2e as vaccines in early stage clinical trials (Deng et al., 2015). Adjuvants may be used to boost the low immunogenicity of conserved sequences (Kang, Kim & Compans, 2012; Lee et al., 2014). Our analysis has shown that H5N1 IAV is highly conserved and contains immune targets that are attractive for vaccine design. Notably, we identified 25 sequences that were highly conserved across both avian and human viruses (≥99% incidence), with half (12) universally conserved to several other subtypes (Heiny et al., 2007); and 12 of the 25 (of which, eight were both pan-subtype conserved and immune-relevant) matched with 30 reported immunogenic human T-cell epitopes, including many that were promiscuous to multiple HLA alleles allowing for greater population coverage. Although, our selection did not result in any targets of HA and NA sequences, which are important for protective immune response (Heiny et al., 2007), the data suggests that they can be selected at a slightly lower, but still highly conserved incidence threshold, such as ≥90%. For example, a reduction of the conservation level to 90% incidence returned nine highly conserved sequences for HA, shared across viruses of both hosts. Further reduction in the conservation threshold (*i.e* to ≥ 80%) is expected to provide even more candidates from HA, which may extend to multiple influenza subtypes, though at a lower conservation level. Careful selection of vaccine targets that protect against a broad spectrum of viral variants and are applicable at the human population is an important first step that is critical for the success of the subsequent steps*.* This work supports the need for knowledge-based approaches towards the design of universal influenza vaccines (Gillim-Ross & Subbarao, 2007).

The study herein also provides data useful for the development of therapeutics and diagnostics, and can aid in the surveillance of existing and future strains of influenza viruses. Highly conserved sequences shortlisted in our analysis could be tested for inhibitory drug design and used as diagnostic tool. There were 68 completely conserved human H5N1 IAV virus sequences (Table S6) that could be used to design targets that are sensitive to detect the presence of H5N1 virus strains. However, these sequences would need to be further evaluated for specificity to the subtype. The high propensity for influenza A virus to develop resistance against drugs has been a major problem for the development of therapeutics against the virus. Rapid viral resistance against current antivirals, M2 ion channel inhibitors (amantadine and rimantadine) and NA inhibitors (oseltamivir, zanamivir, peramivir, and lanimivir) have been reported, with side effects and low efficacy (Yuan, Wen & Zhou, 2018; Zhou et al., 2018). Major research efforts are now focusing on the polymerase protein (such as favipiravir and pimovidir) because they are highly conserved and important machinery in viral replication and transcription (Zhou et al., 2018). Our analyses have shown that there are a number of highly conserved sequences, of non-polymerase protein origin, such as nucleoprotein, neuraminidase and haemaglutinin, which were conserved only in avian viruses. These sequences can be further studied on their presence in other subtypes of H5N1 so that the drug target will have broad effectiveness. Additionally, the diversity motifs can be further assessed by studying the transmission pathway between avian and human virus populations for adaptation patterns. In particular, by assessing for sequences of low incidence motifs (unique, minor or major motifs) in the reservoir that are selected as index in human. For example, a major motif of PB1 (I525V) in avian H7N9 viruses was selected as the index in human viruses (manuscript submitted). The bioinformatics approaches applied herein to study the dynamics of influenza A virus protein sequence diversity can offer a roadmap for a practical, systematic analyses for rationalized vaccine, drug, and diagnostic targets discovery.

## Conclusions

Being an RNA virus, H5N1 IAV is prone to a high mutation rate resulting in a population of related yet distinct viruses, which pose a challenge in vaccine design. This work elucidates and provides important insights on avian and human H5N1 IAV antigenic sequence diversity. Nearly all of the nonamer positions of each viral protein for the two viral hosts had at least one variant sequence; significant variability was observed at certain nonamer positions across both the viral proteomes. Despite the marked variability in the proteome, the majority of the nonamer positions had a highly conserved sequence each, with ≥90% incidence, and the pattern of conservation was grossly similar between the proteins of the avian and human viruses. Consequently, the effects of sequence change to T-cell epitopes were grossly similar between the two viral hosts. Chiefly, analysed nonatypes of both viral hosts were predicted as non-epitopes, indicating that they may escape immune recognition, possibly leading to pathogenesis. Sequence variants of both host populations appear to further support immune evasion with continued sequence change. However, based on our observation, select positions indicate conservation of immune recognition despite significant sequence change, including non-conserved substitutions. Such positions merit further investigation to better understand the pressures that may be subverting immune escape. This study provides a catalogue of all the mutational changes involved in the dynamics of H5N1 IAV diversity for both avian and human host populations. Each of the diversity motifs exhibited a distinctive pattern and together they represented intrinsic patterns in the structure-function organization of the different sequences in H5N1 IAV quasispecies fitness-selection.

Development of an effective vaccine that is able to keep pace with the diversity of the virus is exceptionally challenging. A subtle mutational change may have adverse effect on vaccine efficacy. Hence, highly conserved sequences are thought as a solution in selecting vaccine targets that are representative of the majority of influenza viruses, and raise hope for the possibility of a universal vaccine. Index switching raises concern on the usability of conserved sequences for this purpose. However, only one index switching position was observed for H5N1 IAV among the highly conserved sequences (defined as positions with incidence of ≥80%), and thus, the conserved sequence at this single position can be excluded for vaccine target selection. We expect a similar observation for other IAV subtypes and possibly other viruses, given the constraints to maintain function at highly conserved positions. In summary, this study has demonstrated the presence of H5N1 IAV sequences that are conserved within and between the viruses of the host populations, with some reported as immune targets and exhibiting conservation that goes beyond H5N1 IAV, covering several major subtypes. The selected highly conserved sequences merit further investigation as vaccine targets.

The emergence of new pathogens is alarming. The very recent outbreak (November 2016) of H5N8 (Samuel, Smith & Brown Professor, 2016), H5N6 (World Health Organization, 2016) and H9N2 (World Health Organization, 2019) in many European countries and Asia reminds us of the imminent threat that continued diversification of influenza A viruses poses. Although not every mutation will result in an epidemic or a pandemic, the potential for it to do so should not be underestimated. The key to circumventing this threat is to fully understand the host-pathogen interaction (i.e. immunome). This work is a small step towards that direction.

##  Supplemental Information

10.7717/peerj.7954/supp-1 Figure S1 Sequence diversity motifs The classification of sequences at a given aligned nonamer position as characteristic diversity motifs is shown above for a model nonamer position of 20 sequences. The sequences are ranked according to their incidences. The sequence with the highest incidence (8/20) is classified as the “Index” nonamer, and all others are considered as “Total variants”. The most prevalent sequence among the total variants is classified as the “Major” variant, present in 5 of the 20 isolates. “Minor” variants comprise of sequences that are of lower incidence than the major variant, but are observed more than once; in this case, two distinct sequences, each repeated once, comprised the “Minor” variants (4/20). The three distinct sequences that are observed only once form the “Unique” variants group (3/20). The incidence of “Nonatypes”, which refers to all distinct variant nonamers (6/20), includes one major variant, two minor variants, and three unique variants for this model position. [Adapted from: Hu et al., 2013]

10.7717/peerj.7954/supp-2 Table S1 Country of origin of reported sequences of avian and human influenza A H5N1 viruses reported in Influenza Research Database at the time of data retrieval

10.7717/peerj.7954/supp-3 Table S2 Diversity of avian influenza A (H5N1) virus proteome (raw data) All percentages are shown to the nearest whole number. ^*a*^ Amino acid number at the start and end of the nonamer position in the protein alignment. The symbol # denotes a highly conserved nonamer position (index incidence ≥90%), & denotes a mixed-variable position (index incidence between 90% & 20%), and + denotes a highly diverse nonamer position (index incidence ≤20%). See Fig. S1 for the definition of diversity motifs. ^∗^ Positions with total number of sequences less than 100 ^*b*^ Total number of protein sequences analysed at the aligned nonamer position; the difference in number between the nonamer positions was due to the inclusion of both partial and full-length sequences in the alignments. ^*c*^ Shannon nonamer entropy, which indicates the level of diversity of the nonamer sequences at the position (*H*(*x*); see Fig. 1 for details). ^*d*^ The index nonamer is the most prevalent sequence at the position. ^*e*^ Variants are nonamer sequences that differ by one or more amino acids from the index sequence. ^*f*^ The major variant is the second most common variant sequence at the position. ^*g*^ Minor variants are multiple different repeated nonamer sequences, each occurring more than once and with an incidence of less than or occasionally equal to the major variant. ^*h*^ Unique variants are nonamer sequences that are observed only once at the position. ^*i*^ Nonatypes are distinct sequences among the variants for a given position.

10.7717/peerj.7954/supp-4 Table S3 Diversity of human influenza A (H5N1) virus proteome (raw data) All percentages are shown to the nearest whole number. ^*a*^ Amino acid number at the start and end of the nonamer position in the protein alignment. The symbol # denotes a highly conserved nonamer position (index incidence ≥90%), & denotes a mixed-variable position (index incidence between 90% & 20%), and + denotes a highly diverse nonamer position (index incidence ≤20%). See Fig. S1 for the definition of diversity motifs. ^∗^ Positions with total number of sequences less than 100 ^*b*^ Total number of protein sequences analysed at the aligned nonamer position; the difference in number between the nonamer positions was due to the inclusion of both partial and full-length sequences in the alignments. ^*c*^ Shannon nonamer entropy, which indicates the level of diversity of the nonamer sequences at the position (*H*(*x*); see Fig. 1 for details). ^*d*^ The index nonamer is the most prevalent sequence at the position. ^*e*^ Variants are nonamer sequences that differ by one or more amino acids from the index sequence. ^*f*^ The major variant is the second most common variant sequence at the position. ^*g*^ Minor variants are multiple different repeated nonamer sequences, each occurring more than once and with an incidence of less than or occasionally equal to the major variant. ^*h*^ Unique variants are nonamer sequences that are observed only once at the position. ^*i*^ Nonatypes are distinct sequences among the variants for a given position.

10.7717/peerj.7954/supp-5 Table S4 Immune relevance of index switching positions Only HLA supertypes or alleles with a positive prediction are shown. B cell antigenicity prediction was negative for all the peptides and thus, not shown. Cells in grey shade indicate negative prediction.

10.7717/peerj.7954/supp-6 Table S5 T-cell epitope prediction patterns of sequences from randomly selected nonamer positions of different conservation levels, compared between avian and human hosts. Only prediction for HLA class I was performed Index sequences are in bold, a dot represents similar amino acid as the index sequence, a “-” represents indel. Sequences in red are predicted to be epitopes, whereas those in black are predicted non-epitopes. Cells in yellow indicate sequences shared by both hosts.

10.7717/peerj.7954/supp-7 Table S6 Completely conserved sequences of human Influenza A (H5N1) virus * Positions with total number of sequences less than 100.

10.7717/peerj.7954/supp-8 Data S1 H5N1 human and avian protein alignments

## Additional Information and Declarations

### Data Availability

The following information was supplied regarding data availability: The raw data is available in the Supplemental Files.

## References

[ref-1] Assarsson E, Bui H-H, Sidney J, Zhang Q, Glenn J, Oseroff C, Mbawuike IN, Alexander J, Newman MJ, Grey H, Sette A (2008). Immunomic analysis of the repertoire of T-cell specificities for influenza A virus in humans. Journal of Virology.

[ref-2] Cassone A, Rappuoli R (2010). Universal vaccines: shifting to one for many. mBio.

[ref-3] Chaturvedi S (2009). Pandemic influenza: imminent threat, preparedness and the divided globe. Indian Pediatrics.

[ref-4] Cox NJ, Tamblyn SE, Tam T (2003). Influenza pandemic planning. Vaccine.

[ref-5] Deng L, Cho K, Fiers W, Saelens X, Deng L, Cho KJ, Fiers W, Saelens X (2015). M2e-based universal influenza A vaccines. Vaccine.

[ref-6] Dhanda SK, Usmani SS, Agrawal P, Nagpal G, Gautam A, Raghava GPS (2017). Novel in silico tools for designing peptide-based subunit vaccines and immunotherapeutics. Briefings in Bioinformatics.

[ref-7] Drake JW, Charlesworth B, Charlesworth D, Crow JF (1998). Rates of spontaneous mutation. Genetics.

[ref-8] Dunston CR, Herbert R, Griffiths HR (2015). Improving T cell-induced response to subunit vaccines: opportunities for a proteomic systems approach. Journal of Pharmacy and Pharmacology.

[ref-9] Eigen M, Schuster P (1977). The hypercycle. A principle of natural self-organization. Part A: emergence of the hypercycle. Die Naturwissenschaften.

[ref-10] Evavold BD, Sloan-Lancaster J, Allen PM (1993). Tickling the TCR: selective T-cell functions stimulated by altered peptide ligands. Immunology Today.

[ref-11] Fiers W, De Filette M, Birkett A, Neirynck S, Min Jou W (2004). A universal human influenza A vaccine. Virus Research.

[ref-12] Gillim-Ross L, Subbarao K (2007). Can immunity induced by the human influenza virus N1 neuraminidase provide some protection from avian influenza H5N1 viruses?. PLOS Medicine.

[ref-13] Hasan M, Ghosh PP, Azim KF, Mukta S, Abir RA, Nahar J, Hasan Khan MM (2019). Reverse vaccinology approach to design a novel multi-epitope subunit vaccine against avian influenza A (H7N9) virus. Microbial Pathogenesis.

[ref-14] Heiny AT, Miotto O, Srinivasan KN, Khan MA, Zhang GL, Brusic V, Tan TW, August JT (2007). Evolutionarily conserved protein sequences of influenza a viruses, avian and human, as vaccine targets. PLOS ONE.

[ref-15] Hu Y, Tan PTJ, Tan TW, August JT, Khan MA (2013). Dissecting the dynamics of HIV-1 protein sequence diversity. PLOS ONE.

[ref-16] Huber SKR, Camps MGM, Jacobi RHJ, Mouthaan J, Van Dijken H, Van Beek J, Ossendorp F, De Jonge J (2015). Synthetic long peptide influenza vaccine containing conserved T and B cell epitopes reduces viral load in lungs of mice and ferrets. PLOS ONE.

[ref-17] Jensen KK, Andreatta M, Marcatili P, Buus S, Greenbaum JA, Yan Z, Sette A, Peters B, Nielsen M (2018). Improved methods for predicting peptide binding affinity to MHC class II molecules. Immunology.

[ref-18] Jespersen MC, Peters B, Nielsen M, Marcatili P (2017). BepiPred-2.0: improving sequence-based B-cell epitope prediction using conformational epitopes. Nucleic Acids Research.

[ref-19] Kang SM, Kim MC, Compans RW (2012). Virus-like particles as universal influenza vaccines. Expert Review of Vaccines.

[ref-20] Khan AM, Miotto O, Heiny AT, Salmon J, Srinivasan KN, Nascimento EJM, Marques ETA, Brusic V, Tan TW, August JT (2006). A systematic bioinformatics approach for selection of epitope-based vaccine targets. Cellular Immunology.

[ref-21] Khan AM, Olivo M, Nascimento EJM, Srinivasan KN, Heiny AT, Zhang GL, Marques ET, Tan TW, Brusic V, Salmon J, August JT (2008). Conservation and variability of dengue viru s proteins: implications for vaccine design. PLOS Neglected Tropical Diseases.

[ref-22] Khanna M, Sharma S, Kumar B, Rajput R (2014). Protective immunity based on the conserved hemagglutinin stalk domain and its prospects for universal influenza vaccine development. BioMed Research International.

[ref-23] Kim JH, Skountzou I, Compans R, Jacob J (2009). Original antigenic sin responses to influenza viruses. The Journal of Immunology.

[ref-24] Koo QY, Khan AM, Jung K-O, Ramdas S, Miotto O, Tan TW, Brusic V, Salmon J, August JT (2009). Conservation and variability of West Nile virus proteins. PLOS ONE.

[ref-25] Larsen MV, Lundegaard C, Lamberth K, Buus S, Lund O, Nielsen M (2007). Large-scale validation of methods for cytotoxic T-lymphocyte epitope prediction. BMC Bioinformatics.

[ref-26] Lee Y-T, Kim K-H, Ko E-J, Lee Y-N, Kim M-C, Kwon Y-M, Tang Y, Cho M-K, Lee Y-J, Kang S-M (2014). New vaccines against influenza virus. Clinical and Experimental Vaccine Research.

[ref-27] Meng Z, Han R, Hu Y, Yuan Z, Jiang S, Zhang X, Xu J (2014). Possible pandemic threat from new reassortment of influenza A(H7N9) virus in China. Eurosurveillance.

[ref-28] Miotto O, Heiny AT, Albrecht R, García-Sastre A, Tan TW, August JT, Brusic V (2010). Complete-proteome mapping of human influenza a adaptive mutations: implications for human transmissibility of zoonotic strains. PLOS ONE.

[ref-29] Neumann G, Fujii K, Kino Y, Kawaoka Y (2005). An improved reverse genetics system for influenza A virus generation and its implications for vaccine production. Proceedings of the National Academy of Sciences of the United States of America.

[ref-30] Noda T, Kawaoka Y (2010). Structure of influenza virus ribonucleoprotein complexes and their packaging into virions. Reviews in Medical Virology.

[ref-31] Pan K, Deem MW (2012). Quantifying selection and diversity in viruses by entropy methods, with application to the Hemagglutinin of H3N2 influenza.

[ref-32] Park AW, Wood JLN, Daly JM, Newton JR, Glass K, Henley W, Mumford JA, Grenfell BT (2004). The effects of strain heterology on the epidemiology of equine influenza in a vaccinated population. Proceedings of the Royal Society B: Biological Sciences.

[ref-33] Park MS, Kim J Il, Park S, Lee I, Park M-S (2016). Original antigenic sin response to RNA viruses and antiviral immunity. Immune Network.

[ref-34] Pica N, Palese P (2013). Toward a universal influenza virus vaccine: prospects and challenges. Annual Review of Medicine.

[ref-35] Potter CW (2001). A history of influenza. Journal of Applied Microbiology.

[ref-36] R Core Team (2017). R: A language and environment for statistical computing.

[ref-37] Rammensee H-G (1995). Chemistry of peptides associated with MHC class I and class II molecules. Current Opinion in Immunology.

[ref-38] Saakian DB, Muñoz E, Hu C-K, Deem MW (2006). Quasispecies theory for multiple-peak fitness landscapes. Physical Review. E, Statistical, Nonlinear, and Soft Matter Physics.

[ref-39] Samuel W, Smith J, Brown Professor I (2016). Highly pathogenic avian influenza H5N8 in Europe. https://assets.publishing.service.gov.uk/government/uploads/system/uploads/attachment_data/file/568082/uoa-hpai-europe-20161111.pdf.

[ref-40] Shannon CE (1948). A mathematical theory of communication. Bell System Technical Journal.

[ref-41] Shrestha SS, Swerdlow DL, Borse RH, Prabhu VS, Finelli L, Atkins CY, Owusu-Edusei K, Bell B, Mead PS, Biggerstaff M (2011). Estimating the burden of 2009 pandemic influenza A (H1N1) in the United States (2009–2010). Clinical Infectious Diseases.

[ref-42] Sievers F, Wilm A, Dineen D, Gibson TJ, Karplus K, Li W, Lopez R, McWilliam H, Remmert M, Söding J (2011). Fast, scalable generation of high-quality protein multiple sequence alignments using Clustal Omega. Molecular Systems Biology.

[ref-43] Siegrist C-A, Plotkin SA, Orenstein WA, Offit PA (2013). 2 - Vaccine immunology. Vaccines.

[ref-44] Singh H, Ansari HR, Raghava GPS (2013). Improved method for linear B-cell epitope prediction using antigen’s primary sequence. PLOS ONE.

[ref-45] Sloan-Lancaster J, Allen PM (1996). Altered peptide ligand-induced partial T cell activation: molecular mechanisms and role in T cell biology. Annual Review of Immunology.

[ref-46] Squires RB, Noronha J, Hunt V, García-Sastre A, Macken C, Baumgarth N, Suarez D, Pickett BE, Zhang Y, Larsen CN (2012). Influenza research database: an integrated bioinformatics resource for influenza research and surveillance. Influenza and Other Respiratory Viruses.

[ref-47] Steel J, Lowen AC, Wang TT, Yondola M, Gao Q, Haye K, García-Sastre A, Palesea P (2010). Influenza virus vaccine based on the conserved hemagglutinin stalk domain. mBio.

[ref-48] Szewczyk B, Bieńkowska-szewczyk K, Król E (2014). Introduction to molecular biology of influenza A viruses. Acta Biochimica Polonica.

[ref-49] Taubenberger JK, Kash JC (2010). Influenza virus evolution, host adaptation, and pandemic formation. Cell Host and Microbe.

[ref-50] Taubenberger JK, Morens DM (2006). 1918 Influenza: the mother of all pandemics. Emerging Infectious Diseases.

[ref-51] Uyeki TM (2008). Global epidemiology of human infections with highly pathogenic avian influenza A (H5N1) viruses. Respirology.

[ref-52] Vita R, Mahajan S, Overton JA, Dhanda SK, Martini S, Cantrell JR, Wheeler DK, Sette A, Peters B (2018). The immune epitope database (IEDB): 2018 update. Nucleic Acids Research.

[ref-53] Waterhouse AM, Procter JB, Martin DMA, Clamp M, Barton GJ (2009). Jalview Version 2—a multiple sequence alignment editor and analysis workbench. Bioinformatics.

[ref-54] Webster RG (1998). Influenza: an emerging disease. Emerging Infectious Diseases.

[ref-55] Wickham H (2016). ggplot2: elegant graphics for data analysis.

[ref-56] World Health Organization (2007). A description of the process of seasonal and H5N1 influenza vaccine virus selection and development. Draft 19 November 2007.

[ref-57] World Health Organization (2012). FAQs: H5N1 influenza. http://www.who.int/influenza/human_animal_interface/avian_influenza/h5n1_research/faqs/en/.

[ref-58] World Health Organization (2013). Current WHO phase of pandemic alert (avian influenza H5N1). https://www.who.int/influenza/preparedness/pandemic/h5n1phase/en/.

[ref-59] World Health Organization (2014). H5N1 highly pathogenic avian influenza: timeline of major events. https://www.who.int/influenza/human_animal_interface/H5N1_avian_influenza_update20141204.pdf?ua=1.

[ref-60] World Health Organization (2016). Influenza at the human-animal interface (Summary and assessment, 22 November to 19 December 2016). https://www.who.int/influenza/human_animal_interface/Influenza_Summary_IRA_HA_interface_09_04_2019.pdf.

[ref-61] World Health Organization (2018). Cumulative number of confirmed human cases for avian influenza A(H5N1) reported to WHO, 2003–2018.

[ref-62] World Health Organization (2019). Influenza at the human-animal interface, Summary and assessment, 22 January to 12 February 2019. https://www.who.int/influenza/human_animal_interface/Influenza_Summary_IRA_HA_interface_12_02_2019.pdf?ua=1.

[ref-63] Yuan S, Wen L, Zhou J (2018). Inhibitors of influenza A virus polymerase. ACS Infectious Diseases.

[ref-64] Zhou Z, Liu T, Zhang J, Zhan P, Liu X (2018). Influenza A virus polymerase: an attractive target for next-generation anti-influenza therapeutics. Drug Discovery Today.

[ref-65] Zinkernagel RM, Klenerman P (1998). Original antigenic sin impairs cytotoxic T lymphocyte responses to viruses bearing variant epitopes. Nature.

