# Supplementary figures and images for: Dynamics of Influenza A (H5N1) virus protein sequence diversity
Source: PeerJ. 2020 May 27;7:e7954. doi: 10.7717/peerj.7954 (PMC7261124; doi:10.7717/peerj.7954)

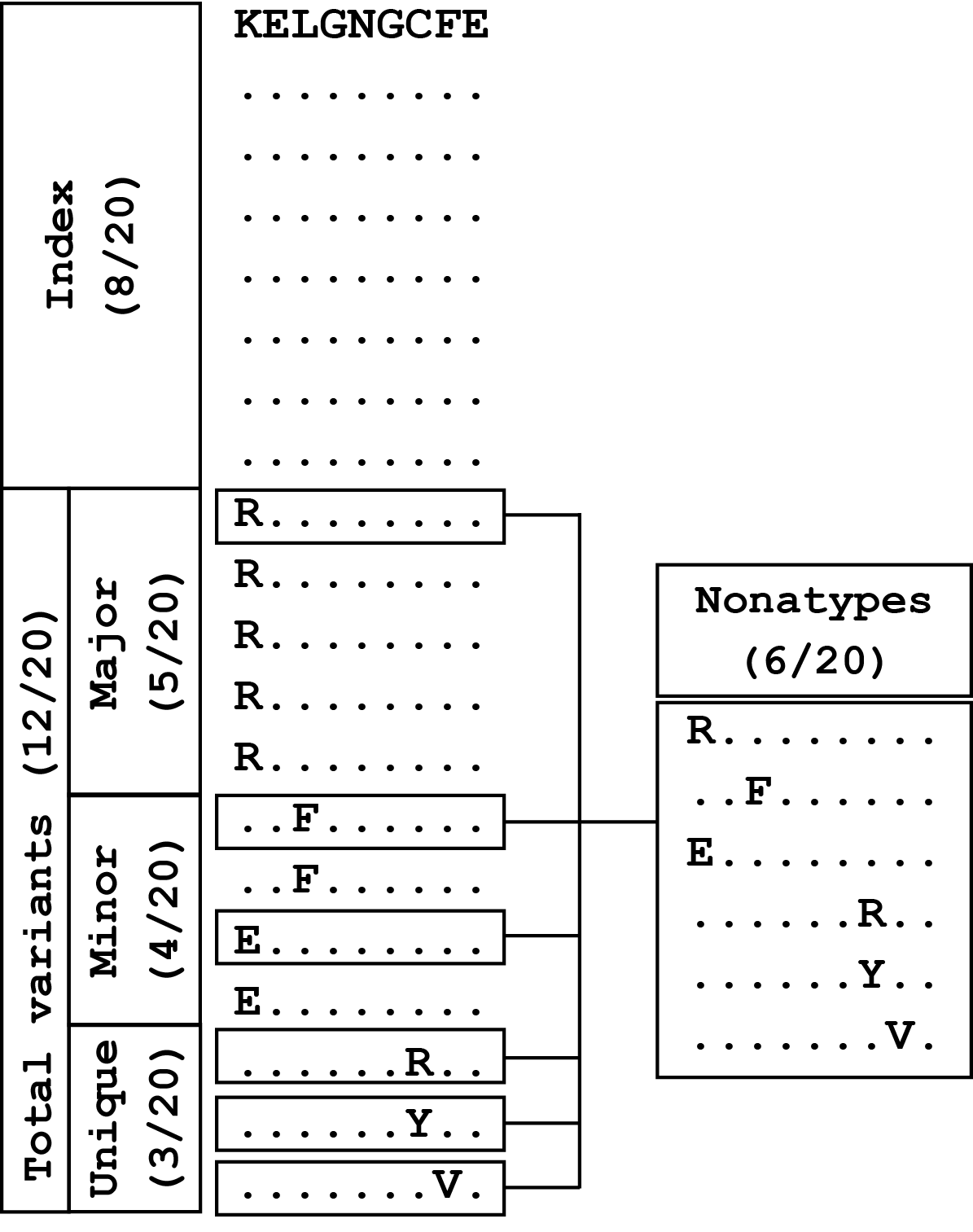

Supplement: Figure S1 — The classification of sequences at a given aligned nonamer position as characteristic diversity motifs is shown above for a model nonamer position of 20 sequences. The sequences are ranked according to their incidences. The sequence with the highest incidence (8/20) is classified as the “Index” nonamer, and all others are considered as “Total variants”. The most prevalent sequence among the total variants is classified as the “Major” variant, present in 5 of the 20 isolates. “Minor” variants comprise of sequences that are of lower incidence than the major variant, but are observed more than once; in this case, two distinct sequences, each repeated once, comprised the “Minor” variants (4/20). The three distinct sequences that are observed only once form the “Unique” variants group (3/20). The incidence of “Nonatypes”, which refers to all distinct variant nonamers (6/20), includes one major variant, two minor variants, and three unique variants for this model position. [Adapted from: Hu et al., 2013] [file peerj-08-7954-s001.png]
