# Supplementary material for: Dynamics of Influenza A (H5N1) virus protein sequence diversity
Source: PeerJ. 2020 May 27;7:e7954. doi: 10.7717/peerj.7954 (PMC7261124; doi:10.7717/peerj.7954)
Supplement: Table S1 [file peerj-08-7954-s002.docx]

**Supplemental Table S1:**

**Country of origin of reported sequences of avian and human influenza A H5N1 viruses reported in Influenza Research Database at the time of data retrieval.**

| Country | Avian | | Human | |
| --- | --- | --- | --- | --- |
|  | Number of reported sequences | Percentage of total (%) | Number of reported sequences | Percentage of total (%) |
| China | 3348 | 29.5 | 235 | 18.3 |
| Viet Nam | 1439 | 12.7 | 209 | 16.3 |
| Egypt | 835 | 7.4 | 156 | 12.1 |
| Hong Kong | 834 | 7.4 | 170 | 13.2 |
| Nigeria | 719 | 6.3 | 2 | 0.2 |
| Thailand | 684 | 6.0 | 72 | 5.6 |
| Azerbaijan | - | - | 20 | 1.6 |
| Indonesia | 619 | 5.5 | 258 | 20.1 |
| Japan | 330 | 2.9 | - | - |
| India | 325 | 2.9 | - | - |
| Laos | 274 | 2.4 | 7 | 0.5 |
| Russia | 237 | 2.1 | - | - |
| Cambodia | 214 | 1.9 | 107 | 8.3 |
| South Korea | 194 | 1.7 | - | - |
| USA | 127 | 1.1 | - | - |
| Germany | 111 | 1.0 | - | - |
| Bangladesh | 93 | 0.8 | 17 | 1.3 |
| Myanmar | 75 | 0.7 | - | - |
| United Kingdom | 70 | 0.6 | - | - |
| Mongolia | 69 | 0.6 | - | - |
| Sweden | 57 | 0.5 | - | - |
| Canada | 50 | 0.4 | - | - |
| Czech Republic | 42 | 0.4 | - | - |
| Italy | 37 | 0.3 | - | - |
| France | 37 | 0.3 | - | - |
| Djibouti | - | - | 1 | 0.1 |
| Burkina Faso | 35 | 0.3 | - | - |
| Denmark | 34 | 0.3 | - | - |
| Pakistan | 33 | 0.3 | - | - |
| Romania | 31 | 0.3 | - | - |
| Saudi Arabia | 25 | 0.2 | - | - |
| Kazakhstan | 24 | 0.2 | - | - |
| Afghanistan | 22 | 0.2 | - | - |
| Austria | 22 | 0.2 | - | - |
| Cote dIvoire | 21 | 0.2 | - | - |
| Turkey | 20 | 0.2 | 18 | 1.4 |
| Sudan | 20 | 0.2 | - | - |
| Hungary | 20 | 0.2 | - | - |
| Israel | 18 | 0.2 | - | - |
| Kuwait | 18 | 0.2 | - | - |
| Malaysia | 18 | 0.2 | - | - |
| Ukraine | 17 | 0.2 | - | - |
| Slovenia | 14 | 0.1 | - | - |
| Bhutan | 14 | 0.1 | - | - |
| Belgium | 11 | 0.1 | - | - |
| Iran | 10 | 0.1 | - | - |
| Poland | 9 | 0.1 | - | - |
| Bulgaria | 9 | 0.1 | - | - |
| Iraq | 9 | 0.1 | 14 | 1.1 |
| Slovakia | 9 | 0.1 | - | - |
| South Africa | 8 | 0.1 | - | - |
| Gaza Strip | 6 | 0.1 | - | - |
| Switzerland | 6 | 0.1 | - | - |
| Bosnia and Herzegovina | 6 | 0.1 | - | - |
| Niger | 5 | 0.0 | - | - |
| Croatia | 4 | 0.0 | - | - |
| Nepal | 3 | 0.0 | - | - |
| Ghana | 2 | 0.0 | - | - |
| Benin | 1 | 0.0 | - | - |
| Mexico | 1 | 0.0 | - | - |
| Togo | 1 | 0.0 | - | - |
| Croatia | 4 | 0.0 | - | - |
