# Supplementary material for: Dynamics of Influenza A (H5N1) virus protein sequence diversity
Source: PeerJ. 2020 May 27;7:e7954. doi: 10.7717/peerj.7954 (PMC7261124; doi:10.7717/peerj.7954)
Supplement: Table S6 — * Positions with total number of sequences less than 100. [file peerj-08-7954-s007.docx]

**Supplemental Table S6:**

**Completely conserved sequences of human Influenza A (H5N1) virus.**

| Protein | Position^a^ | Completely conserved human H5N1 virus sequences |
| --- | --- | --- |
| M1 | *41 - 58 | ALMEWLKTRPILSPLTKG |
| M1 | *60 - 76 | LGFVFTLTVPSERGLQR |
| M1 | *158 - 167 | QHRSHRQMAT |
| M1 | *169 - 183 | TNPLIRHENRMVLAS |
| M1 | *185 - 204 | TAKAMEQMAGSSEQAAEAME |
| M1 | *240 - 248 | YQKRMGVQM |
| NP | 68 - 76 | LSAFDERRN |
| NP | 102 - 116 | GKWVRELILYDKEEI |
| NP | 118 - 127 | RIWRQANNGE |
| NP | 137 - 145 | MIWHSNLND |
| NP | 147 - 155 | TYQRTRALVR |
| NP | 218 - 229 | AYERMCNILKGK |
| NP | 248 - 256 | PGNAEIEDL |
| NP | 258 - 269 | FLARSALILRGS |
| NP | 271 - 282 | AHKSCLPACVYG |
| NP | 290 - 306 | DFEREGYSLVGIDPFRL |
| NP | 385 - 396 | YWAIRTRSG |
| NP | 457 - 465 | SFQGRGVFE |
| NP | 486 - 494 | SYFFGDNAE |
| NS1 | 8 - 16 | SFQVDCFLW |
| NS2 | *101 - 109 | QALQLLLEV |
| PA | 71 - 84 | LLKHRFEIIEGRDR |
| PA | 143 - 154 | THIHIFSFTGEE |
| PA | 172 - 180 | KTRLFTIRQ |
| PA | 242 - 251 | IEGKLSQMSK |
| PA | 426 - 439 | DEIGEDVAPIEHIA |
| PA | 509 - 519 | SHLRNDTDVVN |
| PA | 521 - 532 | VSMEFSLTDPRL |
| PA | 583 - 591 | RCLLQSLQQ |
| PA | 654 - 665 | QLEGFSAESRKL |
| PA | 691 - 711 | EECLINDPWVLLNASWFNSFL |
| PA-X | 71 - 84 | LLKHRFEIIEGRDR |
| PA-X | 143 - 154 | THIHIFSFTGEE |
| PA-X | 164 - 180 | DEESRARIKTRLFTIRQ |
| PB1 | 1 - 11 | MDVNPTLLFLK |
| PB1 | 26 - 43 | GDPPYSHGTGTGYTMDTV |
| PB1 | 78 - 86 | EPSGYAQTD |
| PB1 | 133 - 148 | LNRNQPAATALANTIE |
| PB1 | 159 - 170 | ESGRLIDFLKDV |
| PB1 | 220 - 234 | RALTLNTMTKDAERG |
| PB1 | 236 - 248 | LKRRAIATPGMQI |
| PB1 | 329 - 345 | QPEWFRNVLSIAPIMFS |
| PB1 | 401 - 417 | ASLSPGMMMGMFNMLST |
| PB1 | 476 - 494 | NMSKKKSYINRTGTFEFTS |
| PB1 | 500 - 511 | GFVANFSMELPS |
| PB1 | 537 - 553 | NDLGPATAQMALQLFIK |
| PB1 | 555 - 565 | YRYTYRCHRGD |
| PB1 | 623 - 631 | RLCNPLNPF |
| PB1 | 668 - 676 | PKRNRSILN |
| PB1 | 695 - 703 | LFEKFFPSS |
| PB1-F2 | *52 - 62 | HKQIVYWKQWL |
| PB2 | 30 - 55 | IIKKYTSGRQEKNPALRMKWMMAMKY |
| PB2 | 91 - 104 | VSPLAVTWWNRNGP |
| PB2 | 260 - 267 | IAARNIVRR |
| PB2 | 323 - 331 | SFGGFTFKRT |
| PB2 | 400 - 408 | AMVFSQEDCM |
| PB2 | 430 - 438 | MHQLLRHFQK |
| PB2 | 534 - 548 | MMWEINGPESVLNTY |
| PB2 | 574 - 581 | MEFEPFQSL |
| PB2 | 630 - 642 | MQFSSLTVNVRGSG |
| PB2 | 741 - 751 | SILTDSQTATKR |
| NA | *47 - 62 | EPCNQSIIISNTNFLT |
| NA | 153 - 161 | PSPYNSRFE |
| NA | 355 - 363 | SGFEMIWDP |
| HA | 114 - 124 | YEELKHLLSRI |
| HA | 393 - 402 | GVTNKVNSII |
| HA | 437 - 448 | VWTYNAELLVLM |
| HA | 450 - 461 | NERTLDFHDSNV |

* Positions with total number of sequences less than 100
